# Endogenous Retrovirus-Derived Long Noncoding RNA Enhances Innate Immune Responses via Derepressing RELA Expression

**DOI:** 10.1128/mBio.00937-19

**Published:** 2019-07-30

**Authors:** Bin Zhou, Fei Qi, Fangyi Wu, Hongbo Nie, Yifan Song, Lu Shao, Jingxuan Han, Zhen Wu, Hexige Saiyin, Gang Wei, Penghua Wang, Ting Ni, Feng Qian

**Affiliations:** a State Key Laboratory of Genetic Engineering & MOE Key Laboratory of Contemporary Anthropology, Collaborative Innovation Center of Genetics and Development, Human Phenome Institute, School of Life Sciences and Huashan Hospital, Fudan University, Shanghai, People’s Republic of China; b Ministry of Education Key Laboratory of Contemporary Anthropology, Human Phenome Institute, School of Life Sciences, Fudan University, Shanghai, People’s Republic of China; c Shanghai Vocational College of Agriculture and Forestry, Shanghai, People’s Republic of China; d Department of Immunology, School of Medicine, UConn Health, Farmington, Connecticut, USA; Tufts University; Brown University

**Keywords:** NF-κB, antiviral immune responses, endogenous retroviruses, gene regulation, lncRNA

## Abstract

Endogenous retroviruses (ERVs) are transposable elements that cause host genome instability and usually play deleterious roles in disease such as tumorigenesis. Recent advances also suggest that this “enemy within” may encode a viral mimic to induce antiviral immune responses through viral sensors. Here, through whole-genome transcriptome analysis with RNA sequencing (RNA-Seq), we discovered that a full-length ERV-derived long noncoding RNA (lncRNA), designated lnc-EPAV (ERV-derived lncRNA positively regulates antiviral responses), was a positive regulator of NF-κB signaling. lnc-EPAV expression was rapidly upregulated by viral RNA mimics or RNA viruses to facilitate the expression of RELA, an NF-κB subunit that plays a crucial role in antiviral responses. Transcriptome analysis of lnc-EPAV-silenced macrophages showed that lnc-EPAV was critical for RELA target gene expression and innate immune responses. Consistently, lnc-EPAV-deficient mice exhibited reduced expression of type I interferons (IFNs) and, consequently, increased viral loads and mortality following lethal RNA virus infection. Mechanistically, lnc-EPAV promoted expression of RELA by competitively binding to and displacing SFPQ, a transcriptional repressor of *Rela*. Altogether, our work demonstrates an alternative mechanism by which ERVs regulate antiviral immune responses.

## Abstract (precis)

Endogenous retroviruses are transposable genetic elements comprising 8% to 10% of the human and mouse genomes. Although most ERVs have been inactivated due to deleterious mutations, some are still transcribed. However, the biological functions of transcribed ERVs are largely unknown. Here, we identified a full-length ERV-derived lncRNA, designated lnc-EPAV, as a positive regulator of host innate immune responses. We found that silencing lnc-EPAV impaired virus-induced cytokine production, resulting in increased viral replication in cells. The lnc-EPAV-deficient mice exhibited enhanced susceptibility to viral challenge. We also found that lnc-EPAV regulated expression of RELA, an NF-κB subunit that plays a critical role in antiviral responses. ERV-derived lncRNA coordinated with a transcription repressor, SFPQ, to control *Rela* transcription. Our report provides new insights into the previously unrecognized immune gene regulatory mechanism of ERV-derived lncRNAs.

## INTRODUCTION

Endogenous retroviruses (ERVs) are the result of successful retroviral insertions of ancient and modern retroviruses, which have successfully transferred from parent to progeny by colonizing in the germ line of their hosts (1, 2). Proviruses encode a series of viral proteins (Gag, Pol, and Env) and are flanked by two long terminal repeats (LTRs) which work as regulatory regions for provirus transcription (3, 4). ERVs that retain these typical viral structures are commonly referred to as full-length ERVs (FL-ERVs) (5). Because of the ability to replicate their own DNA, the ERV elements are present at high copy numbers and it is estimated that ERV elements are present at levels of 8% to 10% in the human and mouse genomes (6, 7). The ERVs contribute to gene regulation in both a favorable and an unfavorable manner. On the one hand, some ERVs are detrimental to host fitness and act by inserting proviruses into genome (8). On the other hand, some ERVs promote spatial and temporal expression of host genes (9, 10). Generally speaking, aberrantly activated ERVs and LTRs can be involved in tumorigenesis and tissue development (11). For example, a human ERV-derived gene can activate the extracellular signal–regulated kinase (ERK) pathway and promote expression of transcription factors (TFs) involved in oncogenesis (12). Although most ERVs have lost their retroviral activity, some of them are still transcribed (13, 14). Recently, it has been found that ERVs are significantly enriched in long noncoding RNA (lncRNA) exons relative to protein-coding gene exons (15, 16). These transcribed full-length ERVs are described as FL-ERV-derived lncRNAs. It is estimated that 10% of human endogenous retrovirus subfamily H (HERV-H) transcripts are lncRNAs (16, 17). Accumulating data suggest that regular lncRNAs play an important role in antiviral responses by decoying, scaffolding, or guiding other molecules, such as protein, RNA, and DNA. For example, human THRIL (TNFα and hnRNPL related immunoregulatory LincRNA) and NRAV (negative regulator of antiviral response) and mouse NeST (Nettoie Salmonella pas Theiler’s) regulate antiviral responses by modulating the transcription of the tumor necrosis alpha gene (*TNF-α*), interferon (IFN)-stimulated genes (ISGs) (e.g., *IFITM3* and *MxA*), and the *Ifng* gene (18–20). However, little is known about the role of ERV-derived lncRNAs in antiviral responses.

Host antiviral immunity begins with viral recognition (of, e.g., viral nucleic acids and proteins) by several families of pathogen recognition receptors (PRRs), such as RIG-I-like receptor (RLR), Toll-like receptor (TLR), and NOD-like receptor (NLR) (21). PRRs initiate downstream signaling pathways that lead to activation of downstream transcriptional factors, including nuclear factor kappa-light-chain-enhancer of activated B cells (NF-κB) and interferon regulatory factor 3/7 (IRF3/7), to promote interferon production, which elicits an antiviral state by inducing expression of hundreds of ISGs (22, 23). Recent studies found that expression of bidirectionally transcribed ERVs increased the levels of cytosolic double-stranded RNA (dsRNA), which could be recognized by PRRs, leading to interferon pathway activation (24). However, the role of ERV-derived lncRNAs in viral infection and their molecular mechanisms of action have not been systematically examined.

In this study, we characterized the physiological function of one of the most dramatically upregulated ERV-derived lncRNAs, designated lnc-EPAV (ERV-derived lncRNA positively regulates antiviral responses), which was identified by genome-wide profiling of ERV-derived lncRNA expression in mouse genome. *In vitro* and *in vivo* studies revealed that lnc-EPAV acted as a positive regulator of host antiviral responses through controlling the transcription of *Rela*. It bound and sequestered SFPQ, a transcriptional repressor of *Rela*. In addition, RELA promoted expression of lnc-EPAV, which formed a positive-feedback loop to facilitate antiviral immune responses.

## RESULTS

### Upregulation of ERV-derived lncRNAs in mouse macrophages by viral mimics.

**FIG 1:**
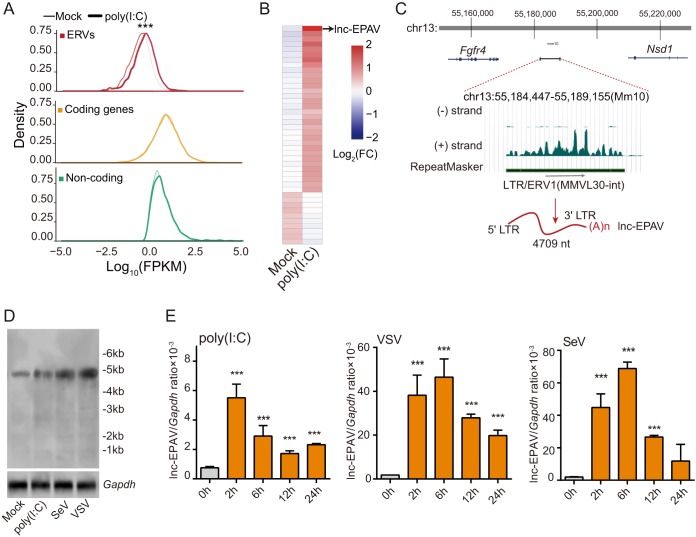
ERV-derived noncoding RNAs are induced by poly(I·C) stimulation in BMDMs. (A) Standardized expression levels (density distribution plots, log10 FPKM) of ERV-derived transcripts, coding genes (NM in RefSeq gene annotation), and known noncoding RNAs (NR in RefSeq gene annotation) in BMDMs treated with 10 μg/ml poly(I·C) for 2 h. ***, *P < *0.001 (Kolmogorov-Smirnov test [KS test]). (B) Heat map of differentially expressed FL-ERVs (FPKM, ≥1; fold change, ≥2) in BMDMs stimulated with poly(I·C) versus mock stimulation. Expression levels are coded in colors ranging from blue (downregulation) to red (upregulation). (C) Schematic diagram of lnc-EPAV. lnc-EPAV is located on chromosome 13qB1 and is flanked by the coding genes *Fgfr4* and *Nsd1* (upper panel). FL-ERV-derived lncRNA lnc-EPAV is transcribed from positive (+) DNA strand (lower panel). (D and E) The lnc-EPAV expression was determined by Northern blotting analysis (D) and qPCR analysis (E) in BMDMs. BMDMs were stimulated with poly(I·C) (10 μg/ml) or infected with SeV or VSV for the indicated times. Data shown represent means ± SEM. ***, *P < *0.001 (Student′s *t* test). Data are representative of results from at least three independent experiments.

To investigate the dynamic transcription of ERV-derived transcripts induced by pathogens, genome-wide transcriptome analysis with RNA sequencing (RNA-seq) was performed for bone marrow-derived macrophages (BMDMs) from C57BL/6 mice stimulated with or not stimulated with viral mimic poly(I·C). Significant increases in the levels of expression of inflammatory cytokines, interferons, and ISGs confirmed activation of the immune responses (see Fig. S1A in the supplemental material). By comparing the global expression patterns of ERVs, we found that the expression levels of ERVs were lower than those of coding genes (NM in RefSeq gene annotation) and known noncoding RNAs (NR in RefSeq gene annotation) in resting cells (Fig. 1A), consistent with the notion that most ERVs are genomic mutants or are silenced by the host due to evolutionary stress (25, 26). Interestingly, expression levels of ERV were globally induced by poly(I·C) stimulation, while those of coding genes and known noncoding RNAs mainly remained unchanged (Fig. 1A), suggesting that ERVs are more sensitive to pathogenic stimuli.

10.1128/mBio.00937-19.1 FIG S1 Poly(I:C)-induced or virus-induced immune responses in BMDMs. (A) Quantification of *Il6*, *Il1b*, *Ifnb1*, and *Ccl5* mRNA levels by qPCR in BMDMs stimulated with poly(I·C) (10 μg/ml) for 2 h. (B) Quantification of lnc-EPAV expression levels by qPCR in BMDMs from BALB/c and 129/Sv mice after VSV infection (MOI = 0.1) for 6 h. Three biological replicates were pooled for qPCR. Data shown represent means ± SEM of results from three technical replicates. ***, *P < *0.001 (Student′s *t* test). Download FIG S1, PDF file, 0.2 MB. Copyright © 2019 Zhou et al. 2019 Zhou et al. This content is distributed under the terms of the Creative Commons Attribution 4.0 International license.

Since FL-ERVs contain complete proviral sequences and likely serve as lncRNAs with comprehensive features when their coding regions are mutated, we next examined the dynamic expression changes of these FL-ERVs upon poly(I·C) treatment in mouse macrophages. We identified 5,322 FL-ERVs among a total of 896,922 ERV elements from the mouse genome by the use of LTR_FINDER (27). To further define the transcribed FL-ERV-derived noncoding RNAs with high confidence, the Coding Potential Assessment Tool (CPAT) algorithm (default coding probability cutoff value of ≤0.44 indicating noncoding sequence) (28) coupled with a strict threshold (uniquely aligned reads, ≥5; fragments per kilobase per million [FPKM] transcripts mapped, ≥0.1 per FL-ERV) was applied. Finally, we identified 1,278 FL-ERV-derived noncoding RNAs among 5,322 FL-ERVs. The corresponding heat map showed that most of the differentially FL-ERV-derived noncoding RNAs (FPKM value of ≥1 and fold change value of ≥2) were rapidly upregulated after stimulation (Fig. 1B), consistent with the trend of global ERV expression shift (Fig. 1A). Among these, a lncRNA of the ERV1 family was found to be the most highly upregulated transcript (Fig. 1B). We named this lncRNA “lnc-EPAV” and characterized its potential functions in antiviral innate immunity. lnc-EPAV was transcribed from the positive strand of the intergenic region flanked by the coding genes *Fibroblast growth factor receptor 4* (*Fgfr4*) and *Nuclear receptor-binding SET-domain protein 1* (*Nsd1*) in the 13qB1 chromosome (Fig. 1C).

We next verified whether lnc-EPAV was upregulated by RNA viruses. Northern blotting detected an ∼4.7-kb lnc-EPAV transcript, in line with the full-length signal identified by RNA-seq (Fig. 1C and D). Importantly, a stronger Northern blot band was observed upon poly(I·C), Sendai virus (SeV), and vesicular stomatitis virus (VSV) stimulation (Fig. 1D), confirming the increased expression of lnc-EPAV stimulated by both pathogenic mimics and viruses. Real-time fluorescence quantitative PCR (qPCR) independently confirmed such upregulation upon pathogenic stimulation (Fig. 1E). To determine whether expression of lnc-EPAV is conserved in different mouse strains, we assessed the transcription level of lnc-EPAV in other commonly used experimental mouse strains. The qPCR results showed that lnc-EPAV was also upregulated in BMDMs from BALB/c and 129/Sv mouse strains after VSV infection (Fig. S1B). All of these results demonstrate that lnc-EPAV expression can be upregulated by both pathogenic mimics and viruses.

### lnc-EPAV is activated by NF-κB subunit RELA.

**FIG 2:**
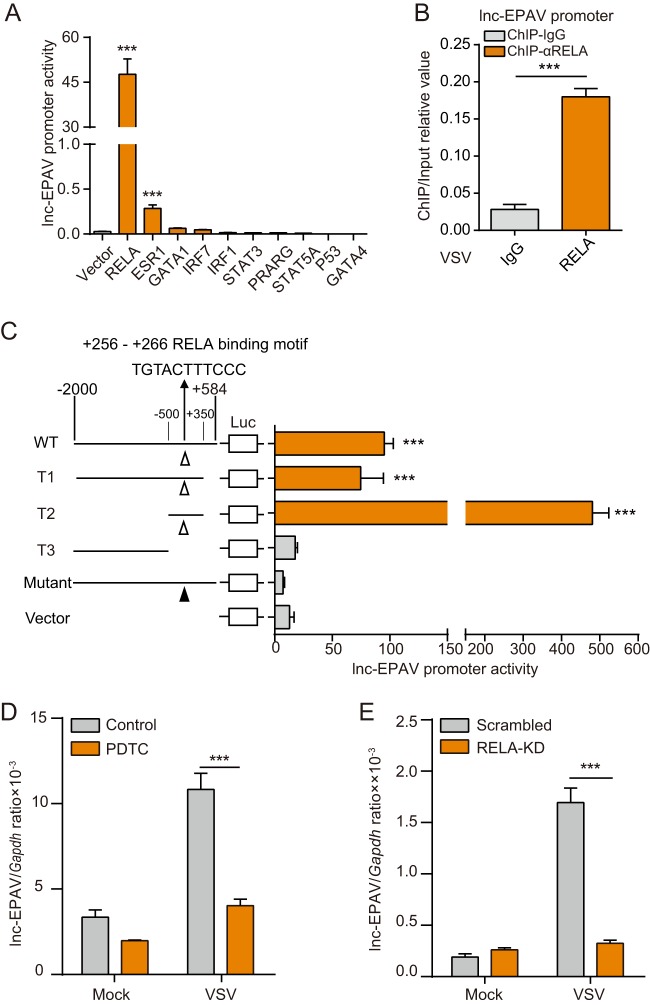
lnc-EPAV is activated by NF-κB subunit RELA. (A) Dual-luciferase assays of lnc-EPAV promoter activity in HEK293T cells transfected with either an empty vector or the indicated transcription factor plasmids. (B) ChIP-qPCR analysis of RELA binding in the lnc-EPAV promoter in BMDMs infected with VSV (multiplicity of infection [MOI] = 0.1) for 6 h. (C) Schematic illustration of truncation and mutation constructs of lnc-EPAV promoter region (nt −2000 to +584 relative to TSS) (left). Quantification of the lnc-EPAV promoter activity in HEK293T cells transfected with various truncated variants of lnc-EPAV promoter together with RELA plasmid (right). (D) qPCR analysis of lnc-EPAV expression from BMDMs pretreated with PDTC (10 μM) for 6 h and then infected with VSV (MOI = 0.1) for 12 h. (E) qPCR analysis of lnc-EPAV expression from J774A.1 macrophages stably expressing either scrambled shRNA or *Rela*-targeting shRNA after VSV infection (MOI = 0.1) for 12 h. KD, knockdown. Data shown represent means ± SEM. ***, *P < *0.001 (Student′s *t* test). Data are representative of results from at least three independent experiments.

We hypothesized that the rapid upregulation of lnc-EPAV after pathogenic stimulation was mediated by immune-related transcription factors (TFs). To identify such TFs, the TRANSFAC database was used to analyze the TF binding sites of promoter region (including the 5′ long terminal repeat [5′ LTR]) of lnc-EPAV. Gene Ontology (GO) annotation enrichment analysis was performed (Fig. S2A), and 10 putative immune system-related TFs in the lnc-EPAV promoter region were selected for further investigation. We assessed the effect of these TFs on lnc-EPAV promoter activation by a luciferase reporter assay. The results showed that overexpression of RELA significantly induced activation of the lnc-EPAV promoter (Fig. 2A). RELA occupancy of lnc-EPAV promoter was also confirmed by chromatin immunoprecipitation-quantitative PCR (ChIP-qPCR) (Fig. 2B). Sequence analysis showed that there is a potential NF-κB/RELA binding motif (at nucleotide [nt] +256 to nt +266 relative to transcription start sites [TSS]) at the lnc-EPAV 5′ LTR region. To characterize the RELA binding motif, we generated a series of lnc-EPAV promoter truncation and mutation constructs for luciferase reporter assay (Fig. 2C, left). Overexpression of RELA induced the activation of lnc-EPAV promoter wild-type (WT), T1, and T2 constructs but failed to activate the T3 and mutant constructs that were devoid of NF-κB/RELA binding motif (5′-TGTACTTTCCC-3′) (Fig. 2C, right). The results of these experiments suggest that the region spanning nt +256 to nt +266 of lnc-EPAV 5′ LTR contains the binding site for RELA-mediated activation.

10.1128/mBio.00937-19.2 FIG S2 lnc-EPAV is activated by NF-κB subunit RELA. (A) GO term enrichment analysis was performed for potential transcription factors involved in regulating lnc-EPAV expression. (B) Confocal immunofluorescence microscopy of RELA in BMDMs pretreated with PDTC (10 μM) for 6 h and then infected with VSV (MOI = 0.1) for 12 h. RELA was stained with a mouse anti-RELA antibody followed by a secondary antibody conjugated with Alexa Fluor 546. Images were acquired using a Carl Zeiss LSM710 microscope (objective, 40×). (C) Immunoblotting analysis of RELA expression in J774A.1 macrophages expressing shRNA against *Rela.* (D) Analysis of the distribution of FL-ERV-derived lncRNAs from four ERV families (ERV1, ERVK, ERVL, and ERVL-MaLR). The number and percentage of lncRNAs of four ERV families are shown in parentheses. (E) Analysis of the RELA binding motif numbers per FL-ERV-derived lncRNA in four ERV families. Download FIG S2, PDF file, 2.3 MB. Copyright © 2019 Zhou et al. 2019 Zhou et al. This content is distributed under the terms of the Creative Commons Attribution 4.0 International license.

To further assess the functional role of RELA in VSV-induced lnc-EPAV expression, BMDMs were treated with NF-κB-specific inhibitor pyrrolidine dithiocarbamate (PDTC), which prevented RELA from transferring to the nucleus and accumulating in the cytoplasm (Fig. S2B). This treatment reduced the level of expression of lnc-EPAV after VSV infection (Fig. 2D). Consistent with this result, the RNA levels of lnc-EPAV were significantly lower in *Rela*-silenced cells than in control cells upon VSV infection (Fig. 2E; see also Fig. S2C). Taken together, these results demonstrate that NF-κB subunit RELA is required for activated transcription of lnc-EPAV.

We next asked whether the RELA motif also existed in other ERV families. Four ERV family-derived lncRNAs were identified in this study, namely, ERV1, ERVL, ERVL-MaLR, and ERVK (Fig. S2D). By scanning the putative ERV-derived lncRNA promoter sequences (the 5′ LTR sequence plus 500 nt before 5′ LTR), we found that the RELA binding motif was globally located in the promoter region of four ERV families (Fig. S2E). However, the average number of RELA motifs in the members of the ERV1 and ERVL-MaLR families was higher than in the members of the ERVL and ERVK families. We speculated that the transcription of lnc-EPAV was controlled by several factors, including TFs and epigenetic modification. RELA is among the key factors that contribute to the upregulation of lnc-EPAV.

### lnc-EPAV enhances cellular antiviral responses.

**FIG 3:**
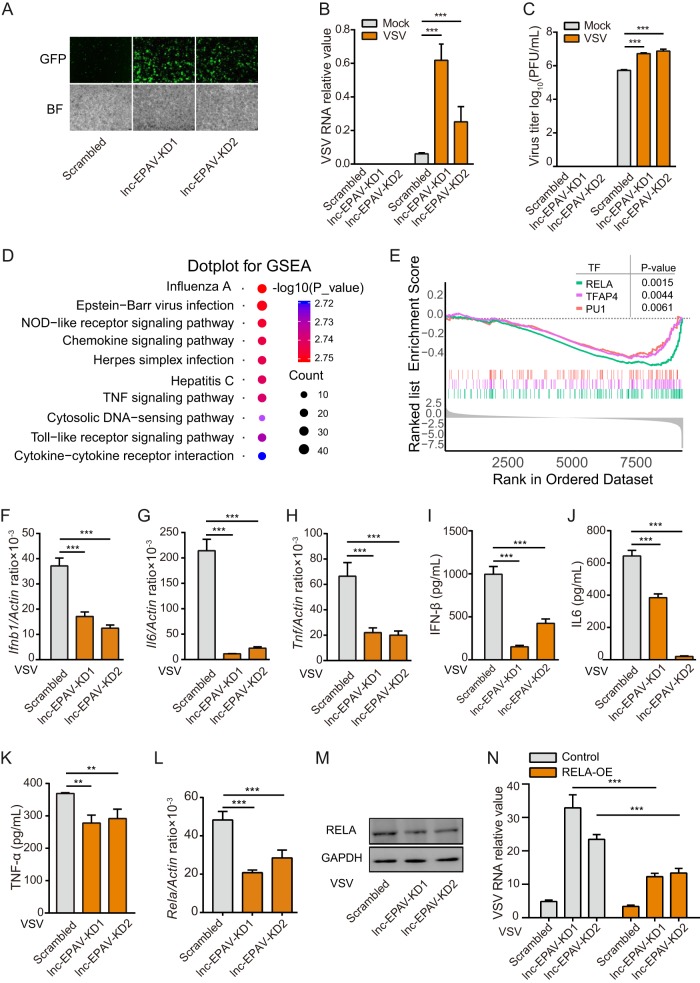
lnc-EPAV positively regulates cellular antiviral responses. (A) Microscopic images of VSV-GFP-infected J774A.1 macrophages stably expressing either scrambled shRNA or lnc-EPAV-targeting shRNA (objective, 5×). BF, bright-field. (B and C) Quantification of intracellular VSV loads by qPCR (B) or of infectious viral particles in the culture medium by plaque assay (C) from J774A.1 macrophages stably expressing either scrambled shRNA or lnc-EPAV-targeting shRNA after VSV infection (MOI = 0.1) for 12 h. (D) GSEA was performed with KEGG gene sets by comparing RNA-seq data between lnc-EPAV knockdown J774A.1 macrophages and control macrophages infected with VSV (MOI = 0.1) for 12 h. Shown are the top 10 significantly enriched KEGG pathways. (E) GSEA was performed with transcription factor target set (MSigDB C3-TFT) in lnc-EPAV knockdown cells versus control cells after VSV infection (MOI = 0.1) for 12 h. (F to H) qPCR quantification of *Ifnb1* (F), *Il6* (G), and *Tnf* (H) expression levels from J774A.1 macrophages stably expressing either scrambled shRNA or lnc-EPAV-targeting shRNA after VSV infection (MOI = 0.1) for 12 h. (I to K) Quantification by type I IFN bioassays or ELISA of secreted IFN-β (I), IL-6 (J), and TNF-α (K) levels from J774A.1 macrophages stably expressing either scrambled shRNA or lnc-EPAV-targeting shRNA after VSV infection (MOI = 0.1) for 12 h. (L and M) qPCR analysis of *Rela* mRNA expression (L) and immunoblot analysis of RELA protein expression (M) from J774A.1 macrophages stably expressing either scrambled shRNA or *Sfpq*-targeting shRNA after VSV infection (MOI = 0.1) for 12 h. (N) Forced expression of RELA could reverse the effects of silencing lnc-EPAV on viral replication. Data represent results of qPCR analysis of intracellular VSV loads from J774A.1 macrophages expressing the indicated shRNAs and expression plasmids after VSV infection (MOI = 0.1) for 12 h. Data shown represent means ± SEM. **, *P < *0.01; ***, *P < *0.001 (Student′s *t* test). Data are representative of results from at least three independent experiments.

To investigate the role of lnc-EPAV in cellular antiviral responses, we designed short hairpin RNAs (shRNAs) targeting two different sites of lnc-EPAV and generated lnc-EPAV-silenced mouse J774A.1 macrophages. Endogenous lnc-EPAV was silenced efficiently as quantified by qPCR (Fig. S3A). We did not observe any off-target effects on shRNA putative target sequences (Fig. S3B to I). Next, we measured the levels of replication of a recombinant VSV expressing green fluorescent protein (VSV-GFP) in lnc-EPAV-silenced cells. Silencing lnc-EPAV greatly enhanced VSV replication in terms of GFP-positive (GFP^+^) cell numbers (Fig. 3A). Consistent with this, both the viral RNA levels measured by qPCR and the virus titers determined by plaque assay showed that silencing lnc-EPAV significantly promoted viral replication in J774A.1 macrophages (Fig. 3B and C). In addition, replication of VSV was dramatically attenuated by lnc-EPAV overexpression (Fig. S3J). These data suggest that lnc-EPAV is involved in cellular antiviral responses.

10.1128/mBio.00937-19.3 FIG S3 lnc-EPAV enhances cellular antiviral responses. (A) Quantification of endogenous lnc-EPAV expression by qPCR from J774A.1 macrophages stably expressing either scrambled shRNA or lnc-EPAV-targeting shRNA. (B to I) Quantification of putative shRNA off-target ERV transcript levels by qPCR from J774A.1 macrophages stably expressing either scrambled shRNA or lnc-EPAV-targeting shRNA. (J) Quantification of intracellular VSV loads by qPCR from J774A.1 macrophages overexpressing lnc-EPAV after VSV infection (MOI = 0.1) for 12 h. Data shown represent means ± SEM. ***, *P < *0.001 (Student′s *t* test). Data are representative of results from at least three independent experiments. Download FIG S3, PDF file, 0.5 MB. Copyright © 2019 Zhou et al. 2019 Zhou et al. This content is distributed under the terms of the Creative Commons Attribution 4.0 International license.

To explore the underlying mechanism by which lnc-EPAV modulates antiviral responses, we performed RNA-seq to analyze the global effects of lnc-EPAV in J774A.1 macrophages infected with VSV for 12 h. A total of 16 significant pathways were identified in lnc-EPAV-silenced cells through gene set enrichment analysis (GSEA) performed with KEGG gene sets (normalized enrichment scores [NES] greater than or equal to 1 or less than or equal to −1; false-discovery-rate [*q*] value, ≤0.05). Most enriched KEGG pathways were involved in pathogen infection and immune responses (Fig. 3D). GSEA was performed with the transcription factor target set (MSigDB C3-TFT) and identified NF-κB/RELA as a master transcription factor associated with the immune responses in lnc-EPAV knockdown cells (Fig. 3E). Consistently, the expression levels of of NF-κB/RELA target genes, including the beta interferon (IFN-β), interleukin-6 (IL-6), and TNF-α genes, significantly decreased at both the mRNA level (Fig. 3F to H) and the protein level (Fig. 3I to K) in lnc-EPAV knockdown cells after VSV infection. These results implied the presence of cross talk between NF-κB/RELA and lnc-EPAV.

We next evaluated the impact of lnc-EPAV knockdown on RELA expression. Depletion of endogenous lnc-EPAV significantly reduced *Rela* expression (Fig. 3L). Immunoblotting confirmed downregulation of RELA protein levels in lnc-EPAV-silenced cells (Fig. 3M). We hypothesized that lnc-EPAV might regulate antiviral responses through upregulation of RELA and, if so, that forced expression of RELA could reverse the effects of silencing lnc-EPAV on viral replication. To this end, exogenous RELA was overexpressed in the lnc-EPAV-silenced J774A.1 macrophages. Overexpression of RELA rescued the effects of silencing lnc-EPAV to inhibit VSV replication (Fig. 3N). These results suggest that lnc-EPAV may regulate the expression of RELA and its target genes during virus infection and may consequently inhibit viral replication.

### SFPQ is a binding partner of lnc-EPAV in the nucleus.

**FIG 4:**
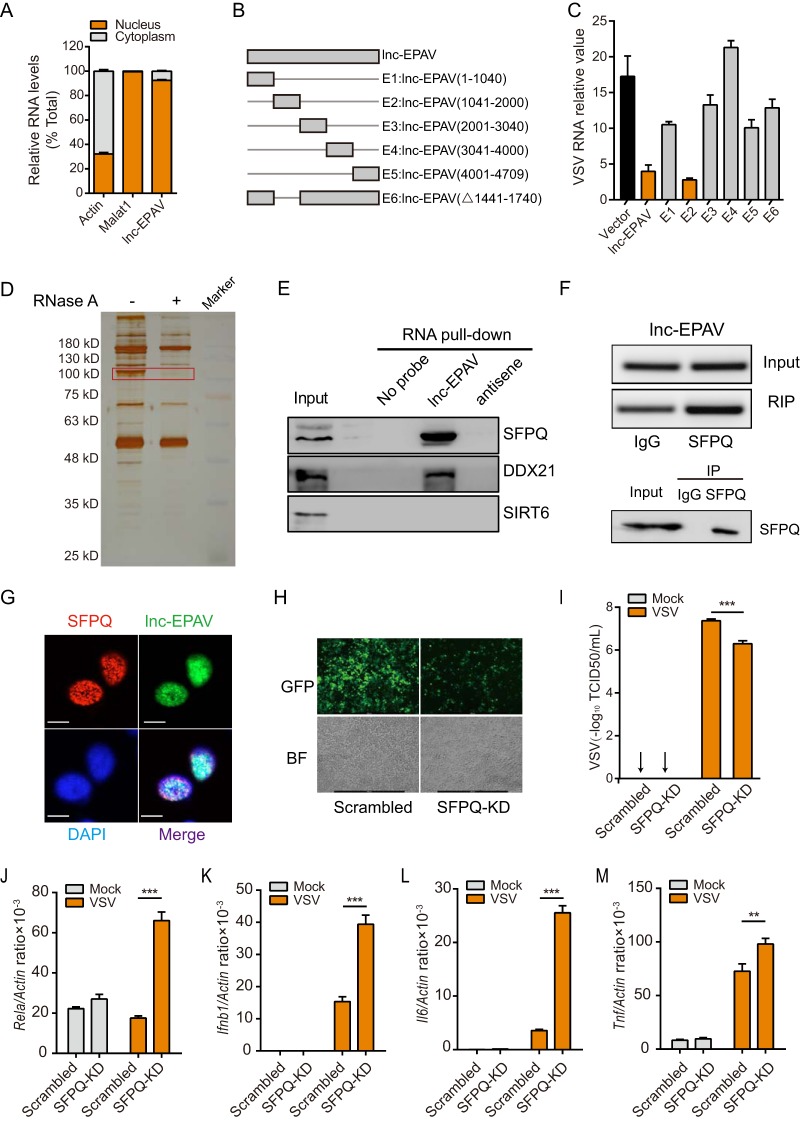
Identification of SFPQ as a binding protein of lnc-EPAV. (A) qPCR of lnc-EPAV expression levels between nuclear and cytoplasmic compartments from BMDMs. Equivalent amounts of nuclear and cytoplasmic RNAs were used as the templates. (B) Schematic diagram of various truncation and deletion mutations of lnc-EPAV. (C) Quantification by qPCR of intracellular VSV loads from J774A.1 macrophages overexpressing lnc-EPAV or its truncation and deletion mutations after VSV infection (MOI = 0.1) for 12 h. (D) Silver staining of biotinylated lnc-EPAV-associated proteins. The lnc-EPAV-specific bands (highlighted bands) were excised and analyzed by mass spectrometry. (E) Immunoblots of proteins from RNA pulldown assay by biotinylated lnc-EPAV or antisense RNA. SIRT6 was used as a negative control. (F) SFPQ RIP followed by RT-PCR analysis of copurified RNAs from non-cross-linked BMDMs. (G) RNA FISH detecting endogenous lnc-EPAV (green) combined with immunofluorescence staining of SFPQ (red) in BMDMs. DAPI staining is shown in blue. Bar, 10 μM. (H) Microscopic images of VSV-GFP-infected SFPQ-knockdown J774A.1 macrophages (objective, 5×). (I) Quantification of infectious VSV particles in the culture medium by 50% tissue culture infective dose (TCID_50_) assay from J774A.1 macrophages stably expressing either scrambled shRNA or *Sfpq*-targeting shRNA, after VSV infection (MOI = 0.1) for 12 h. BF, bright-field. (J to M) qPCR analysis of *Rela* (J), *Ifnb1* (K), *Il6* (L), and *Tnf* (M) expression from J774A.1 macrophages stably expressing either scrambled shRNA or *Sfpq*-targeting shRNA after VSV infection (MOI = 0.1) for 12 h. Data shown represent means ± SEM. **, *P < *0.01; ***, *P < *0.001 (Student′s *t* test). Data are representative of results from at least three independent experiments.

Although we have provided clues indicating that RELA was a key regulator in mediating lnc-EPAV-dependent antiviral effects, the details of the molecular mechanism by which lnc-EPAV controls RELA expression are still lacking. qPCR of nuclear fractions and of cytoplasmic fractions revealed that lnc-EPAV was mostly located in the nucleus (Fig. 4A). These data hint that lnc-EPAV executed its function in the nucleus. To characterize the functional region of lnc-EPAV, we constructed a series of lnc-EPAV truncation constructs (Fig. 4B). Each lnc-EPAV truncation mutant was overexpressed in J774A.1 macrophages and was then assessed for its antiviral effects. Full-length lnc-EPAV and the E2 lnc-EPAV truncation mutant were found to affect the virus replication most significantly (Fig. 4C). These results suggest that RNA sequences (1,041 to 2,000 nt; E2) of lnc-EPAV are essential for its function.

As nuclear lncRNAs usually interact with proteins to exert their functions, we applied RNA pulldown coupled with mass spectrometry (MS) to identify the interacting proteins of lnc-EPAV. Biotinylated full-length lnc-EPAV and lnc-EPAV E2 truncation mutants were incubated with nuclear extracts and pulled down with streptavidin magnetic beads. The associated proteins were analyzed by SDS-PAGE with silver staining (Fig. 4D; see also Fig. S4A) followed by mass spectrometry. By analyzing the full-length and E2 region sequences of lnc-EPAV interacting proteins, we identified two potential RNA binding proteins, namely, SFPQ and DDX21 (see Table S1 in the supplemental material; see also data available under ProteomeXchange identifier PXD011577). To confirm the binding of these proteins to lnc-EPAV, we first performed an independent RNA pulldown experiment. The results showed that the sense strand of lnc-EPAV bound both SFPQ and DDX21 but that the antisense strand failed to do so (Fig. 4E). Next, we asked if endogenous SFPQ and DDX21 were able to coimmunoprecipitate with lnc-EPAV. Only the anti-SFPQ antibody enriched lnc-EPAV and not the anti-DDX21 antibody (Fig. 4F; see also Fig. S4B). These complementary experiments suggested that SFPQ was a physiological binder of lnc-EPAV. Moreover, RNA fluorescence *in situ* hybridization (RNA-FISH) combined with immunofluorescence further demonstrated the colocalization of lnc-EPAV and SFPQ in the nucleus of BMDMs (Fig. 4G).

10.1128/mBio.00937-19.4 FIG S4 SFPQ is a binding partner of lnc-EPAV in the nucleus. (A) Silver staining of biotinylated lnc-EPAV-E2-associated proteins. The lnc-EPAV-E2 specific bands (highlighted bands) were excised and analyzed by mass spectrometry. (B) DDX21 RIP followed by RT-PCR analysis of copurified RNAs from non-cross-linked BMDMs. (C) Immunoblotting analysis of RELA expression in J774A.1 macrophages stably expressing shRNA against *Sfpq*. Download FIG S4, PDF file, 2.8 MB. Copyright © 2019 Zhou et al. 2019 Zhou et al. This content is distributed under the terms of the Creative Commons Attribution 4.0 International license.

10.1128/mBio.00937-19.6 TABLE S1 Mass spectrometry analysis of SFPQ. Download Table S1, DOCX file, 0.02 MB. Copyright © 2019 Zhou et al. 2019 Zhou et al. This content is distributed under the terms of the Creative Commons Attribution 4.0 International license.

Many lncRNAs are known to interact with nuclear proteins (e.g., TFs and RNA binding proteins) to regulate gene expression (29, 30). SFPQ is a nuclear protein with DNA and RNA binding activity and exerts transcriptional inhibition of *CYP17* (31) and *IL-8* (32). We speculated that lnc-EPAV may cooperate with SFPQ to regulate downstream immune gene expression. To test this, we knocked down *Sfpq* by the use of shRNA, which led to reduced viral replication in terms of GFP^+^ cell numbers (Fig. 4H) and VSV titers (Fig. 4I). In line with a reduction in VSV loads, SFPQ knockdown resulted in increased levels of mRNA expression of immune genes, including *Rela*, *Ifnb1*, *Il6*, and *Tnf* (Fig. 4J to M), and in increased levels of RELA protein (Fig. S4C). These results indicate that the binding of lnc-EPAV to SFPQ may derepress the transcription activity of immune genes and ultimately contribute to antiviral effects.

### lnc-EPAV cooperates with SFPQ to regulate *rela*.

**FIG 5:**
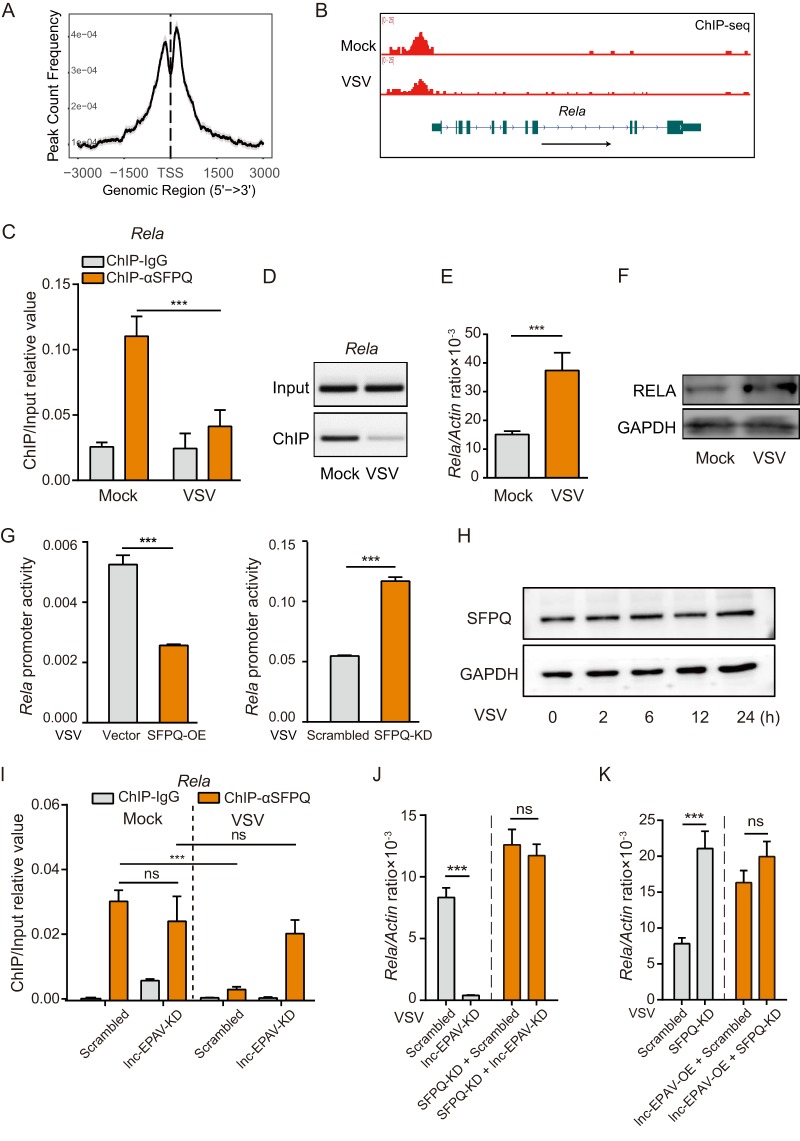
lnc-EPAV cooperates with SFPQ to regulate *Rela*. (A) The distribution of putative SFPQ binding sites was enriched around the TSS gene (−3 kb to +3 kb around TSS). (B) Gene tracks software (Integrated Genome Browser) was used for ChIP-seq analysis of SFPQ enrichment at the promoter region of *Rela* in BMDMs with or without VSV infection. (C and D) ChIP-qPCR analysis (C) and ChIP-PCR analysis (D) of SFPQ in the *Rela* promoter in BMDMs infected with VSV (MOI = 0.1) for 12 h. (E) qPCR analysis of *Rela* mRNA expression level in BMDM after VSV infection (MOI = 0.1) for 6 h. (F) Immunoblot analysis of RELA protein expression level in BMDM after VSV infection (MOI = 0.1) for 12 h. (G) Dual-luciferase assays of *Rela* promoter activity in SFPQ-overexpressing cells (SFPQ-OE; left) or SFPQ-knockdown cells (SFPQ-KD; right) after VSV infection (MOI = 0.1) for 12 h. (H) Immunoblotting analysis of SFPQ protein in BMDMs infected with VSV (MOI = 0.1) for the indicated times. (I) ChIP-qPCR analysis of SFPQ in the *Rela* promoter in J774A.1 macrophages stably expressing either scrambled shRNA or lnc-EPAV-targeting shRNA after VSV infection (MOI = 0.1) for 12 h. (J) qPCR analysis of *Rela* expression level in J774A.1 macrophages expressing the indicated shRNA after VSV infection (MOI = 0.1) for 12 h. (K) qPCR analysis of *Rela* expression level in J774A.1 macrophages expressing the indicated shRNA or lnc-EPAV overexpression vectors after VSV infection (MOI = 0.1) for 12 h. Data shown represent means ± SEM. ns, not significant; ***, *P < *0.001 (Student′s *t* test). Data are representative of results from at least three independent experiments.

We further explored the details of the mechanism by which lnc-EPAV interacts with SFPQ to regulate antiviral responses. To examine whether SFPQ directly bound to the promoter region of immune genes such as *Rela*, chromatin immunoprecipitation followed by deep sequencing (ChIP-seq) was performed in BMDMs. Model-based ChIP-seq analysis (MACS) (33) was used to detect the statistically significant peaks of mapped reads. The distribution of putative SFPQ binding sites around the TSS gene was enriched (Fig. 5A). We then applied GO term enrichment analysis of the SFPQ putative target genes by ChIP assay and found that 172 were immune genes, including *Rela* (Table S2). To investigate whether SFPQ occupied the promoter region of *Rela* in resting macrophages and ceased to occupy the region after viral infection, we examined the SFPQ representative read coverage over the *Rela* promoter. Notably, a high level of binding signal of SFPQ was observed around the promoter region of *Rela* but the level was attenuated in macrophages after VSV stimulation (Fig. 5B). Such a change of occupancy upon VSV infection was confirmed by ChIP-qPCR and ChIP-PCR (Fig. 5C and D). Meanwhile, the mRNA level of *Rela* was significantly increased after VSV infection (Fig. 5E). Immunoblotting confirmed the upregulation of RELA protein expression in VSV-infected cells (Fig. 5F). We further assessed the effect of SFPQ on *Rela* promoter repression using luciferase reporters. Transient overexpression of SFPQ inhibited the transcriptional activity of *Rela*, while knockdown of SFPQ activated its transcriptional activity after VSV infection (Fig. 5G). Several studies showed that the VSV matrix (M) protein may shut down host cell translation (34, 35). In order to examine whether SPFQ translation was shut down by VSV infection, the level of expression of SFPQ was quantified by immunoblot analysis. The level of protein expression of SFPQ was unchanged during the VSV infection within 24 h (Fig. 5H). These results indicate that the dissociation of SFPQ from *Rela* promoter may promote the transcriptional activation of *Rela* upon viral infection.

10.1128/mBio.00937-19.7 TABLE S2 ChIP-seq peak annotation of immune genes. Download Table S2, XLSX file, 0.02 MB. Copyright © 2019 Zhou et al. 2019 Zhou et al. This content is distributed under the terms of the Creative Commons Attribution 4.0 International license.

To explore whether lnc-EPAV functions through SFPQ, we performed ChIP-qPCR on lnc-EPAV-silenced macrophages. The results of ChIP-qPCR showed that the levels of avidity of SFPQ for *Rela* promoter DNA in the resting state wer esimilar in lnc-EPAV-silenced cells and control cells (Fig. 5I, left). After VSV infection, a significant decrease in SFPQ binding to the *Rela* promoter was observed in control cells, indicating activation of *Rela* transcription (Fig. 5I, Mock versus VSV-infected scrambled control cells). However, the level of SFPQ binding to the *Rela* promoter in lnc-EPAV-silenced cells before and after VSV infection remained the same (Fig. 5I, Mock versus VSV-infected lnc-EPAV-silenced cells). These results indicated that the absence of lnc-EPAV hindered the dissociation of SFPQ from *Rela* promoter under conditions of viral infection, leading to transcriptional repression. Next, we examined whether the positive effect of lnc-EPAV on *Rela* transcription is dependent on SFPQ. The results demonstrated that depletion of lnc-EPAV significantly reduced the *Rela* mRNA expression level but that the effect was absent from SFPQ knockdown cells (Fig. 5J). Consistently, lnc-EPAV overexpression promoted *Rela* expression, whereas it had no effect in SFPQ knockdown cells (Fig. 5K), indicating that lnc-EPAV acts upstream of SFPQ. Altogether, these results suggest that lnc-EPAV binds SFPQ and removes its occupancy in the *Rela* promoter, leading to transcription of *Rela*.

### lnc-EPAV protects mice against viral infection.

**FIG 6:**
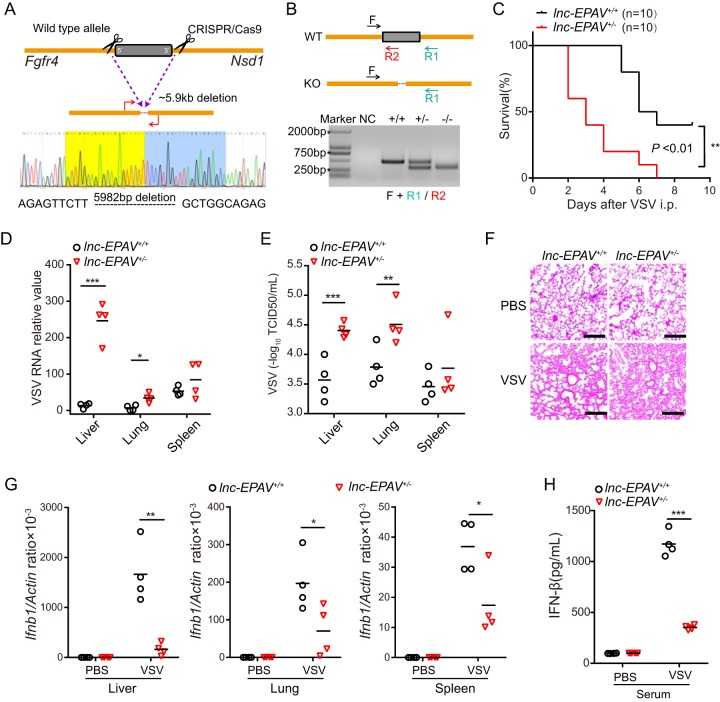
lnc-EPAV protects mice against viral infection. (A) Schematic diagram of CRISPR/Cas9 knockout strategies at lnc-EPAV loci. A deletion of 5,982 bp was confirmed by Sanger sequencing. (B) Genotyping of lnc-EPAV knockout (KO) mice. Genomic DNA PCR products were derived from wild-type, monoallelic-deletion, or biallelic-deletion mice. NC, negative control. (C) Survival of 6-to-8-week-old *lnc-EPAV^+^*^/^*^+^* or *lnc-EPAV^+^*^/−^ mice (*n* = 10 mice per group) after intraperitoneal (i.p.) injection of VSV (5 × 10^7^ plaque forming units [PFU] per mouse). **, *P < *0.01 (log rank test). (D and E) qPCR analysis of VSV RNA (D) and TCID_50_ assay of VSV particles (E) in the liver, lung, and spleen of *lnc-EPAV^+^*^/^*^+^* and *lnc-EPAV^+^*^/−^ mice infected with VSV (5 × 10^7^ PFU per mouse) via intraperitoneal injection for 48 h. *, *P < *0.05; **, *P < *0.01; ***, *P < *0.001 (Student′s *t* test). (F) Hematoxylin-and-eosin staining of sections of lungs from mice processed as described for panel B. Bars, 50 μm. PBS, phosphate-buffered saline. (G) qPCR analysis of *Ifnb1* expression in the liver (left panel), lung (center panel), and spleen (right panel) from mice as in B. *, *P < *0.05; **, *P < *0.01 (Student′s *t* test). (H) IFN-β protein levels in serum from mice processed as described for panel B. ***, *P* < 0.001 (Student′s *t* test).

The aforementioned *in vitro* results provided a solid basis for *in vivo* studies. We thus created mice that lost lnc-EPAV by removing the full-length lnc-EPAV genomic locus using clustered regularly interspaced short palindromic repeat (CRISPR)/Cas9 genome-editing technology (Fig. 6A and B). Homozygous female *lnc-EPAV^−^*^/^*^−^* mice exhibited growth deficiency due to unknown reasons, so we chose heterozygous mice and their littermates for experimentation. We challenged *lnc-EPAV^+^*^/^*^+^* and *lnc-EPAV^+^*^/−^ mice with VSV and found that the overall survival rate of the *lnc-EPAV^+^*^/−^ mice was much lower (Fig. 6C). VSV replication levels and titers were significantly higher in the liver and lung of *lnc-EPAV^+^*^/−^ mice than in those from *lnc-EPAV^+^*^/^*^+^* mice (Fig. 6D and E), and there was more infiltration of inflammatory cells into the lungs of *lnc-EPAV^+^*^/−^ mice following infection (Fig. 6F). In addition, the infected *lnc-EPAV^+^*^/−^ mice developed more-severe neurological symptoms as well as decreased movement and limb paralysis in comparison to the *lnc-EPAV^+^*^/^*^+^* mice on day 3 or 4 postinfection. The levels of *Ifnb1* mRNA expression in liver, lung, and spleen of *lnc-EPAV^+^*^/−^ mice were decreased after infection (Fig. 6G). In agreement with this, the level of IFN secretion induced by VSV infection was much lower in serum of *lnc-EPAV^+^*^/−^ mice than in that of *lnc-EPAV^+^*^/^*^+^* mice (Fig. 6H). Collectively, these data indicate that lnc-EPAV is an important positive regulator of antiviral immune responses *in vivo*.

## DISCUSSION

Sequences derived from ERVs constitute a substantial fraction of human and mouse genomes. However, the biological roles of ERVs are still poorly understood. In particular, involvement of any full-length ERV-derived lncRNAs in host immune responses has not yet been reported. In this study, we demonstrated that an ERV-derived lncRNA (named lnc-EPAV) functioned as a positive regulator of virus-induced host antiviral immune responses. lnc-EPAV expression was rapidly upregulated by viral RNA mimics or RNA viruses. Transcriptome analysis of lnc-EPAV-silenced macrophages showed that lnc-EPAV was critical for induction of NF-κB/RELA target genes during viral infection. lnc-EPAV deficiency led to reduced interferon production, resulting in enhanced susceptibility to VSV infection in mice. Mechanically, the expression of lnc-EPAV competitively displaced SFPQ from the *Rela* promoter to release its inhibitory effect, resulting in upregulation of RELA, which in turn promoted the expression of lnc-EPAV in a positive-feedback manner. This work revealed, for the first time, that ERV-derived lncRNA could enhance innate immune responses through derepressing a key immune gene, *Rela*.

Normally, due to evolutionary pressure, ERVs have been inactivated by accumulation of point mutations, insertions, or deletions to avoid deleterious impacts in host genome. The shutdown of ERV activity can also be achieved by epigenetic repression, including that resulting from DNA methylation and histone modifications. To counteract these silencing effects, ERVs hijack host transcription factors to their LTR regions. The LTR region plays a vital role as it contains all the transcriptional elements, including the TATA box, enhancers, and transcription factor binding sites, which are required for initiation of transcription of ERVs (36). In this study, an NF-κB/RELA binding site was identified in the LTR of lnc-EPAV. Some studies estimated that nearly 15% of coding regions simultaneously work as both exon sequence and TF recognition sites (37). Overexpression of RELA significantly induced the activation of lnc-EPAV, whereas silencing of RELA had the opposite effect on virus-induced lnc-EPAV expression. Recruiting RELA to the ERV LTR region may help lnc-EPAV utilize host immune signaling and facilitate its transcription. In addition, we analyzed the key antiviral innate immune response transcription factor binding sites in the LTR region of the 32 upregulated FL-ERV-derived lncRNAs shown in the heat map in Fig. 1B. We found that three representative lncRNAs from different ERV families contained RELA, IRFs, and E74-like ETS transcription factor 4 (ELF4) binding sites (38). These results indicated that other factors might be involved in the regulation of ERV expression. We speculated that the transcription of lnc-EPAV was controlled by several factors, including *trans*-acting factors (e.g., TFs, epigenetic modification) and *cis*-regulatory elements in promoter or LTR regions (39). For example, ERV activation upon loss of histone methylation occurring in a lineage-specific manner depends on specific sets of transcription factors available to LTR regions (40). Therefore, we hypothesized that TFs and epigenetic modifications may work together to regulate the expression of lnc-EPAV.

lncRNAs cooperate with other molecules, usually proteins, to exert their regulatory functions. For example, lnc-DC, NRON, and lncRNA-ACOD1 interact with STAT3, NFAT, and GOT2, respectively (41–43). In this study, SFPQ was identified as a lnc-EPAV-interacting protein involved in antiviral innate immune responses. We investigated whether the SFPQ binding motif (44) in lnc-EPAV was also present in other ERV families. Interestingly, the SFPQ binding motif was specifically present in members of the ERV1 family rather than in those of other ERV families (e.g., EFVK, ERVL, and ERVL-MaLR).

SFPQ is a multifunctional protein that is involved in various biological processes, including paraspeckle function, RNA splicing, intron retention, miRNA synthesis, virus replication, and transcription regulation (45–47). Here we showed that SFPQ acted as a transcriptional repressor of key immune gene *Rela*. In agreement with our findings, it has been reported that SFPQ can also repress the transcription of immune genes such as *IL-8* (32). SFPQ protein belongs to a conserved family of *Drosophila* behavior human splicing (DBHS) proteins (48). DBHS proteins encompass two RNA recognition motif domains (RRM1 and RRM2) to interact with lncRNA. SFPQ can also bind to DNA through its DNA binding domain (DBD) (48, 49). These properties provide a molecular basis for the use of SFPQ by lnc-EPAV to regulate *Rela* expression in the nucleus.

The human and mouse SFPQ proteins share 95.25% identity, which implies conserved function. By analyzing public human SFPQ ChIP-seq data (GSE58444) (50), we observed an enriched distribution of reads around the TSS. Interestingly, we found a strong SFPQ-bound peak at the *RELA* promoter (see Fig. S5A in the supplemental material). By ChIP-qPCR and ChIP-PCR, we experimentally confirmed that SFPQ bound to the promoter region of *RELA* and that the occupancy of SFPQ at the *RELA* promoter was reduced upon VSV infection in human HEK293T cells (Fig. S5B). Consistent with the phenotype of SFPQ knockdown mouse cells, the expression levels of RELA were increased in SFPQ knockdown human cells (Fig. S5C). We hypothesize that human ERV (HERV)-derived lncRNAs may cooperate with human SFPQ to exert function although ERV-derived lncRNAs are not conserved in different species (51). We used RNA immunoprecipitation coupled with deep sequencing (RIP-seq) to examine whether SFPQ bound human ERV-derived transcripts in nuclei. By scanning 506,566 human ERV loci (ERV length, ≥200 nt) from the RepeatMasker database with strict cutoff values (fold change, ≥ 3; FPKM, ≥1), we identified 1,025 putative SFPQ-bound human ERV-derived transcripts in the nucleus (see Table S3 in the supplemental material). The protein-RNA binding between SFPQ and three representative transcribed HERVs (MER9a2, LTR5A, and MLT2A1) was validated with independent RIP followed by reverse transcription-PCR (RT-PCR) (Fig. S5D and E). These findings indicated the biological importance and evolutionary prevalence of such a regulatory mechanism. So our current understanding is that although lnc-EPAV is not evolutionary conserved, interactions of SPFQ with ERV-derived lncRNAs is conserved between mouse and human.

10.1128/mBio.00937-19.5 FIG S5 Human SFPQ regulates RELA expression. (A) ChIP-seq analysis of SFPQ in human MCF cells by using public data (GSE58444). The distribution of putative SFPQ binding sites was enriched around the TSS gene (−2.5 kb to +2.5 kb around the TSS) in MCF cells (left). Gene tracks software (Integrated Genome Browser) was used for analysis of SFPQ enrichment at the promoter region of *RELA* in human MCF cells (right). (B) ChIP-qPCR analysis (left) and ChIP-PCR analysis (right) of SFPQ in the *RELA* promoter in HEK293T cells infected with VSV (MOI = 0.1) for 12 h. (C) qPCR analysis (left) and immunoblotting analysis (right) of RELA expression levels in SFPQ-knockdown HEK293T cells. (D) RIP-seq analysis of putative SFPQ binding HERV-derived transcripts in HEK293T cells (MER9a2, LTR5A, and MLT2A1; normalized density plot). IgG was used as a negative control. (E) SFPQ RIP analysis followed by RT-PCR analysis of copurified RNAs from non-cross-linked HEK293T. Download FIG S5, PDF file, 1.5 MB. Copyright © 2019 Zhou et al. 2019 Zhou et al. This content is distributed under the terms of the Creative Commons Attribution 4.0 International license.

10.1128/mBio.00937-19.8 TABLE S3 Putative SFPQ-bound HERV-derived transcripts. Download Table S3, XLSX file, 0.1 MB. Copyright © 2019 Zhou et al. 2019 Zhou et al. This content is distributed under the terms of the Creative Commons Attribution 4.0 International license.

**FIG 7:**
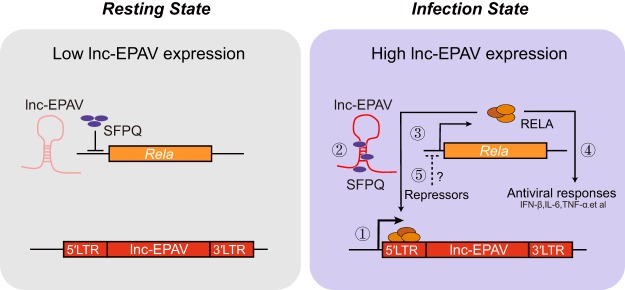
Model of ERV-derived lncRNA in the regulation of antiviral immune responses via RELA. (Left panel) In the resting state, lnc-EPAV was expressed at a low level. SFPQ acted as a transcriptional repressor of key immune gene *Rela*. (Right panel) After virus infection, lnc-EPAV expression was rapidly upregulated and lnc-EPAV was caused to accumulate in the nucleus by the activation of NF-κB/RELA. lnc-EPAV promoted the transcription of *Rela* by competitively binding to and displacing SFPQ, which forms a positive-feedback loop to enhance the antiviral immune responses. After host cells eliminated the infected virus, some putative repressors negatively regulated the activation of RELA, consequently reducing the expression of lnc-EPAV.

The NF-κB transcription factor has vital roles in cellular processes involved in immune responses, inflammation, and oncogenesis (52–54). Although results of many studies investigating regulation of NF-κB/RELA activity through several posttranslational modifications, including acetylation, phosphorylation, and ubiquitination, have been reported previously (55–57), regulation at the transcriptional level is still poorly understood. Here we report that an ERV-derived lncRNA coordinated with a transcription repressor SFPQ to control *Rela* transcription. In turn, RELA promoted the transcription of lnc-EPAV to form a positive-feedback loop (Fig. 7). Our findings regarding lnc-EPAV offer an insight into the previously unrecognized immune regulatory mechanism of ERV-derived lncRNAs.

## MATERIALS AND METHODS

### Antibodies, reagents, and viruses.

Mouse anti-SFPQ (B92; catalog no. P2860), rabbit anti-RELA (Ab-276; catalog no. SAB4300295), and rabbit anti-SFPQ (catalog no. PLA0181) were purchased from Sigma-Aldrich (MO, USA). Mouse anti-RELA (L8F6; catalog no. 6956), rabbit anti-SIRT6 (D8D12; catalog no. 12486), and rabbit anti-GAPDH (anti-glyceraldehyde-3-phosphate dehydrogenase) (D16H11; catalog no. 5174) were purchased from Cell Signaling Technology (MA, USA). Mouse anti-DDX21 (D-8; catalog no. sc-376953) was purchased from Santa Cruz Biotechnology (CA, USA). Alexa Fluor 546-conjugated goat anti-mouse secondary antibody (catalog no. A-11003) was obtained from Thermo Fisher (MA, USA). The antibodies were diluted 1,000 times for immunoblots and 200 times for immunofluorescence and immunoprecipitation. Lipofectamine 2000 (Invitrogen, USA) was used for transfection of nucleic acids. PDTC (catalog no. P8765) was purchased from Sigma-Aldrich (MO, USA). Alexa Fluor 488-conjugated Avidin (catalog no. A-21370) was purchased from Thermo Fisher (MA, USA). High-molecular-weight poly(I·C) (catalog no. tlrl-pic) and puromycin (catalog no. ant-pr-1) were obtained from InvivoGen (USA). Sendai virus (SeV) was a kind gift from Bo Zhong (Wuhan University, China). Green fluorescent protein-tagged vesicular stomatitis virus (VSV-GFP) and VSV provided by Guang Yang (Jinan University, China) were passaged once in Vero cells, and viral PFU levels were quantified by plaque assay (58).

### Plasmid construction.

For nuclear expression of lnc-EPAV, pZW1-snoVector (Addgene plasmid catalog no. 73174) was modified into pCDH-puro (System Biosciences, USA). The full-length or truncated forms of lnc-EPAV were cloned into modified pCDH-pZW1-snoVector. For gene knockdown, the annealed shRNA oligonucleotides for lnc-EPAV, *Sfpq*, and *Rela* were inserted into pLKO.1 vector (Addgene plasmid catalog no. 8453). For overexpression of SFPQ, mouse *Sfpq* was amplified by PCR, cut by restriction enzymes, and inserted into pcDNA3.1-Flag vector. pRL-TK plasmid and pGL3-Basic vector were purchased from Promega for reporter assays. The lnc-EPAV promoter (full-length, truncated, and mutant forms) and *Rela* promoter were cloned into pGL3-Basic vector. All constructs were verified by sequencing the relevant regions. The PCR primers are listed in Table S4 in the supplemental material.

10.1128/mBio.00937-19.9 TABLE S4 Primer information. Download Table S4, DOCX file, 0.02 MB. Copyright © 2019 Zhou et al. 2019 Zhou et al. This content is distributed under the terms of the Creative Commons Attribution 4.0 International license.

### Mouse models.

lnc-EPAV knockout (KO) mice were generated with CRISPR/Cas9 technology on a C57BL/6J background by Biocytogen (Beijing, China). Putative single guide RNA (sgRNA) off-target sequences in mice genome were predicted by the use of Cas-OFFinder tools (59) (http://www.rgenome.net/cas-offinder/) with a cutoff mismatch value of ≤3. The putative off-target sequences are listed in Table S5. We did not observe off-target mutation in these regions by Sanger sequencing. All mice were housed in a specific-pathogen-free (SPF) environment at Fudan University. We used 6-week-old to 8-week-old sex-matched mice for all experiments. The mice were infected with 5 × 10^7^ PFU of VSV through intraperitoneal injection. Morbidity and mortality were monitored twice a day. All mouse experiments were conducted in accordance with the recommendations in the Guide for the Care and Use of Laboratory Animals of Fudan University, with the approval of the Fudan University Laboratory Animal Center (201802148S).

10.1128/mBio.00937-19.10 TABLE S5 Analysis of putative sgRNA off-target sequences. Download Table S5, DOCX file, 1.4 MB. Copyright © 2019 Zhou et al. 2019 Zhou et al. This content is distributed under the terms of the Creative Commons Attribution 4.0 International license.

### Cell culture.

HEK293T cells and Vero cells were obtained from the Type Culture Collection of the Chinese Academy of Science. The J774A.1 macrophages and the L929-ISRE cell line were kind gifts from Guang Yang (Jinan University, China). BMDMs were differentiated using a previously published method (60). The cells were cultured at 37°C under 5% CO_2_ in Dulbecco’s modified Eagle’s medium (DMEM) or RPMI 1640 medium supplemented with 10% fetal bovine serum (FBS) and antibiotics (100 units/ml penicillin and 100 μg/ml streptomycin; Invitrogen).

### Identification and analysis of full-length ERV.

The UCSC (University of California, Santa Cruz) genome browser bioinformatic RepeatMasker (61) and BLAST querying (62) tools were used to identify the ERV elements from mouse genome (version Mm9). The full-length ERV sequences were identified by the use of the LTR_FINDER tool (27).

### RNA-Seq and data analysis.

RNA-seq libraries were prepared according to the instructions provided with KAPA stranded RNA-Seq kits (Kapa Biosystems, USA). The libraries were sequenced using an Illumina HiSeq X Ten platform in a paired-end 2 × 150-bp manner. Processed raw data were aligned to the mouse genome (version Mm9) or human genome (version Hg38) using STAR (63). To analyze the levels of gene expression, the estimated expression levels were converted to FPKM data by the use of Cuffdiff (64).

### Quantitative PCR (qPCR) analysis.

Total RNA was extracted using TRI reagent (Sigma, USA), and cDNA was synthesized using a reverse transcription reagent kit (TaKaRa, Japan). Amplification was performed using SYBR green qPCR master mix (Biotools, China) and gene-specific primers in a CFX-96 system (Bio-Rad, USA), and values were normalized to those of a housekeeping gene. The qPCR primers are listed in Table S4.

### Immunoblotting and Northern blotting.

For immunoblotting, cells were harvested using radioimmunoprecipitation assay (RIPA) lysis buffer containing protease inhibitor cocktail (Biotechwell, China). Equal amounts of proteins were separated by SDS-PAGE and then transferred onto polyvinylidene difluoride (PVDF) membranes (Thermo Fisher, USA). Immunoblots were probed with the indicated antibodies developed by the use of NcmECL Ultra reagent (NCM Biotech, China). For Northern blotting, total RNA of BMDMs was extracted using TRI reagent (Sigma, USA). Biotin-labeled antisense and sense RNA probes (300 bp, 1,441 to 1,740 nt) were made *in vitro* using a HiScribe T7 Quick high-yield RNA synthesis kit (NEB, United Kingdom) and biotin RNA labeling mix (Roche, Germany). The assay was carried out according to the manufacturer’s protocol (NorthernMax kit; Thermo Fisher, USA).

### Dual-luciferase reporter assays.

HEK293T cells were transfected with luciferase reporter plasmids (lnc-EPAV promoter-Luc or *Rela*-Luc), pRL-TK plasmid, or the indicated TF expression plasmid for 24 h. For experiments examining viral infection, cells were infected with VSV (multiplicity of infection [MOI] = 0.1) for 12 h. Cells were lysed, and luciferase activities were measured by the use of a TransDetect dual-luciferase reporter assay kit (Transgene, China) according to the manufacturer’s instructions.

### ELISA and type I interferon bioassays.

Commercial enzyme-linked immunosorbent assay (ELISA) kits were used to measure the levels of IL-6 protein (catalog no. 431301) and TNF-α protein (catalog no. 430901; BioLegend, USA) in cell culture supernatants. The level of type I interferon was measured as described previously (38) with reference to recombinant mouse IFN-β (catalog no. 8234-MB; R&D Systems, USA) as a standard and with L929 cells stably transfected with an interferon sequence response element (ISRE) luciferase construct.

### RNA pulldown assay and RNA immunoprecipitation (RIP).

Biotin-labeled RNA probes lnc-EPAV and lnc-EPAV-E2 (960 bp [1,041 to 2,000 nt]) were made *in vitro* using a HiScribe T7 Quick high-yield RNA synthesis kit (NEB, United Kingdom) and biotin RNA labeling mix (Roche, Germany). Biotinylated probes were incubated with BMDM nuclear extracts (Beyotime, China) for 4 h at room temperature followed by incubation with Dynabeads MyOne Streptavidin C1 beads (Thermo Fisher, USA) at 4°C for 4 h. The proteins on the beads were separated by SDS-PAGE followed by silver staining and mass spectrometry (MS) identification.

Mouse anti-SFPQ antibody, mouse anti-DDX21 antibody, or IgG control was added to the BMDM nuclear extracts and incubated at 4°C for 4 h followed by incubation with SureBeads protein G magnetic beads (Bio-Rad, USA) at 4°C for 2 h. RNA/protein complexes were immunoprecipitated, and RNA was extracted and quantified with RT-PCR.

### Fluorescence *in situ* hybridization and immunofluorescence microscopy.

For lnc-EPAV RNA FISH assay, BMDMs cultured on poly-l-lysine-coated coverslips were fixed with 4% paraformaldehyde, permeabilized with 0.5% Triton X-100, washed, and stored with 70% ethanol at −20°C. Endogenous biotin signal was blocked by using an endogenous biotin blocking kit (Thermo Fisher, USA). BMDMs were incubated with biotin-labeled lnc-EPAV probe at 50°C overnight. The cells were then incubated with Alexa Fluor 488-conjugated avidin (Thermo Fisher, USA) at room temperature for 4 h. For immunofluorescence analysis, cells were sequentially fixed with 4% paraformaldehyde, permeabilized with 0.5% Triton X-100, blocked with 5% bovine serum albumin (BSA), and incubated with primary antibody (mouse anti-SFPQ; Sigma, USA) followed by Alexa Fluor 546-conjugated goat anti-mouse secondary antibody (Thermo Fisher, USA). Nuclei were counterstained with DAPI (4',6-diamidino-2-phenylindole). Images were acquired using a Carl Zeiss LSM710 microscope (objective, 40×).

### ChIP-qPCR, ChIP-seq, and data analysis.

BMDMs were fixed with 1% formaldehyde and quenched with glycine. Purified chromatin was sonicated to a level of 300 to 500 bp using ultrasonic processing (Scientz-IID, China) and incubated with mouse anti-RELA antibody (CST, USA), mouse anti-SFPQ antibody (Sigma, USA), or mouse IgG control (Abmart, China). DNA-protein complexes were immunoprecipitated by the use of salmon sperm DNA-blocked SureBeads protein G magnetic beads (Bio-Rad, USA), followed by reverse cross-linking processes. The DNA was then purified and quantified by qPCR and PCR.

For ChIP-seq library construction, purified DNA was processed for end repair, followed by a 3′-end dA-adding reaction and Y-shape adaptor ligation. The final sequencing library was obtained by PCR amplification, and sequencing was then performed via the use of an Illumina HiSeq X Ten platform. For data analysis, model-based analysis of ChIP-seq (MACS) was used to identify enrichment regions (33). The ChIPseeker R package was used for ChIP peaks annotation and visualization (65).

### Statistical analysis.

GraphPad Prism software was used for statistical analyses. The analyses of results were performed with a two-tailed unpaired Student′s *t* test. Survival curves were analyzed using a log rank (Mantel-Cox) test. Data are presented as means ± standard errors of the means (SEM). Data are representative of results from at least three independent experiments. *P* values of <0.05 were considered statistically significant.

### Data availability.

All sequencing data have been deposited in the NCBI SRA database under accession number PRJNA503657. Data from the proteomics studies are available via ProteomeXchange with identifier PXD011577.
