# Supplementary figures and images for: Endogenous Retrovirus-Derived Long Noncoding RNA Enhances Innate Immune Responses via Derepressing RELA Expression
Source: mBio. 2019 Jul 30;10(4):e00937-19. doi: 10.1128/mBio.00937-19 (PMC6667616; doi:10.1128/mBio.00937-19)

A

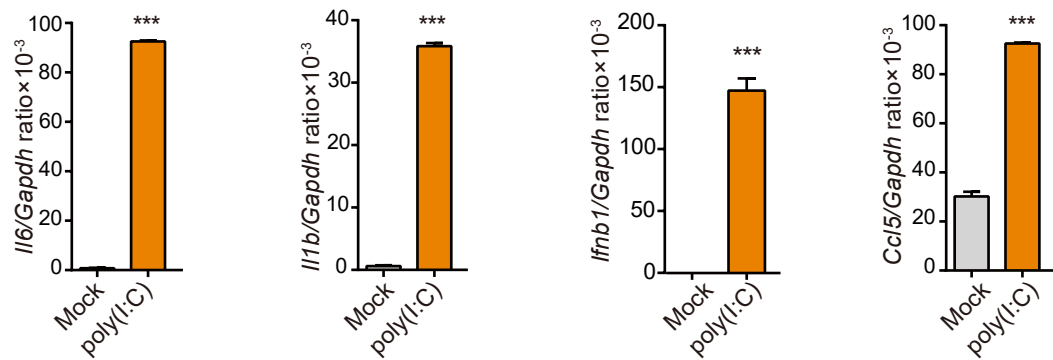

B

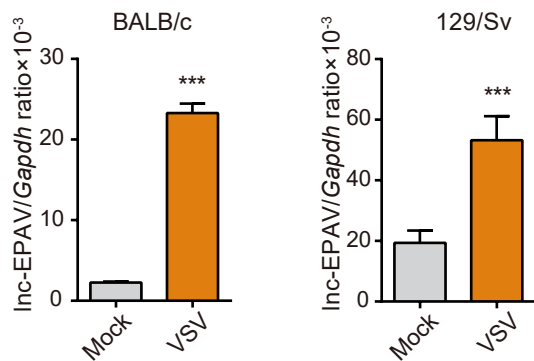

Supplement: FIG S1 [file mBio.00937-19-sf001.pdf]

A

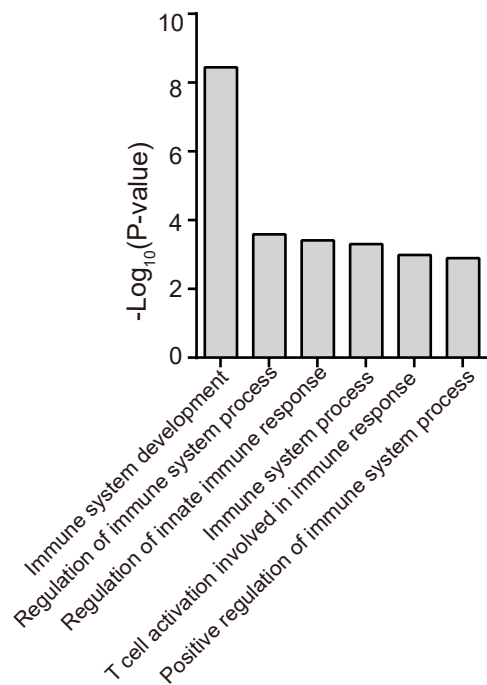

B

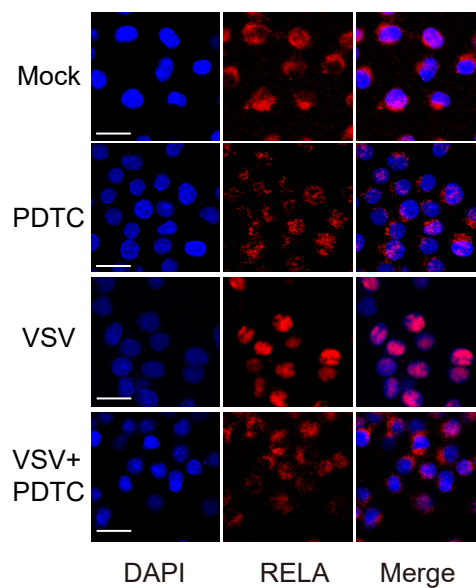

C

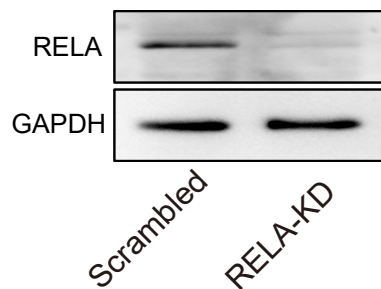

D

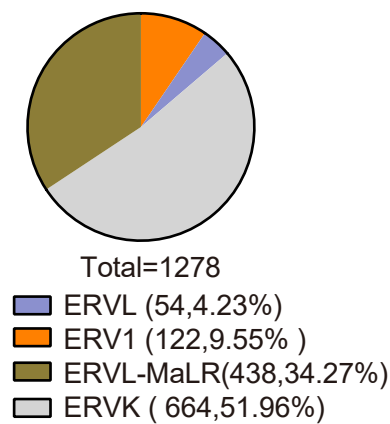

E

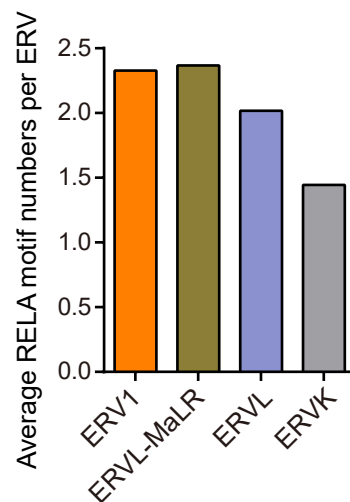

Supplement: FIG S2 [file mBio.00937-19-sf002.pdf]

A

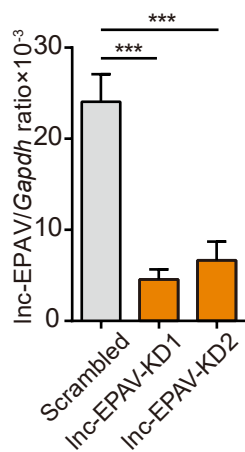

B

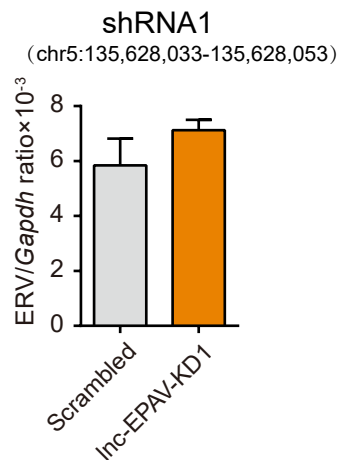

C

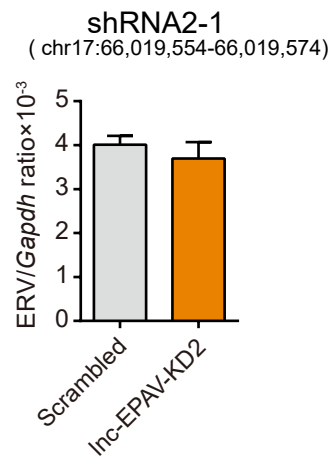

D

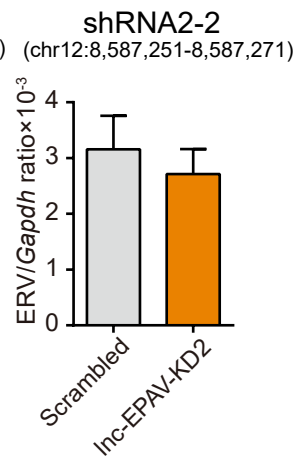

E

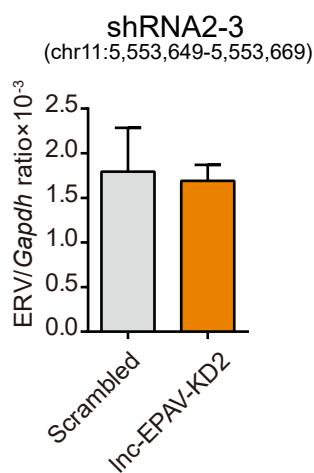

F

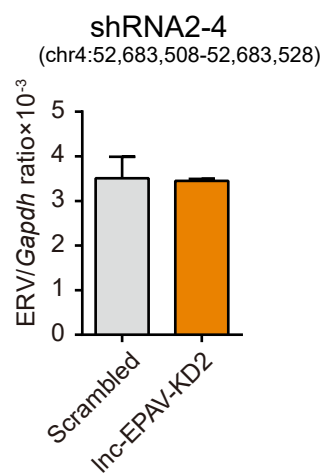

G

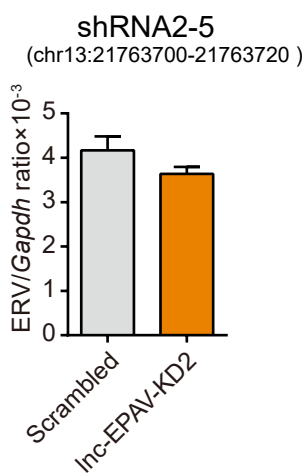

H

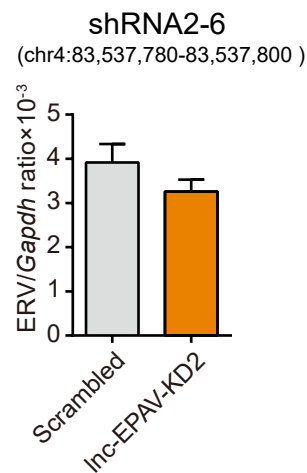

I

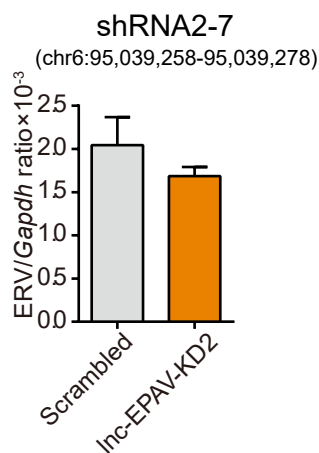

J

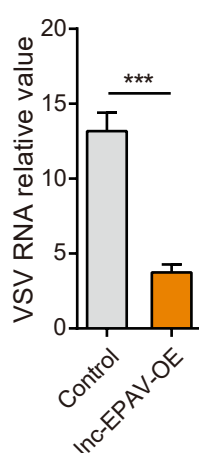

Supplement: FIG S3 [file mBio.00937-19-sf003.pdf]

A

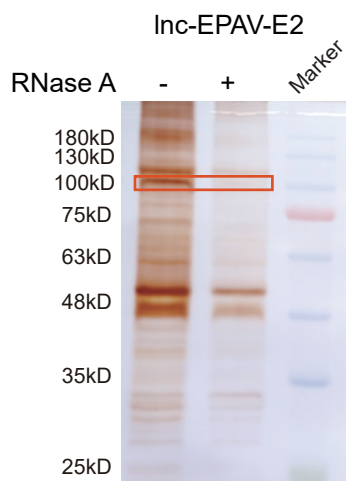

B

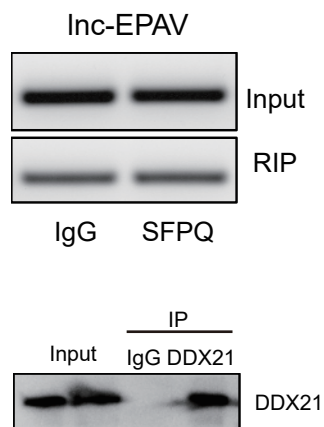

C

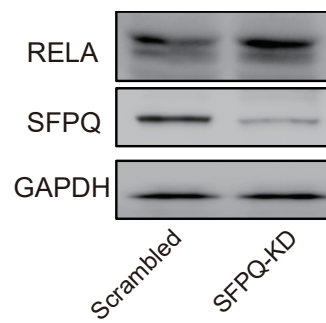

Supplement: FIG S4 [file mBio.00937-19-sf004.pdf]

A

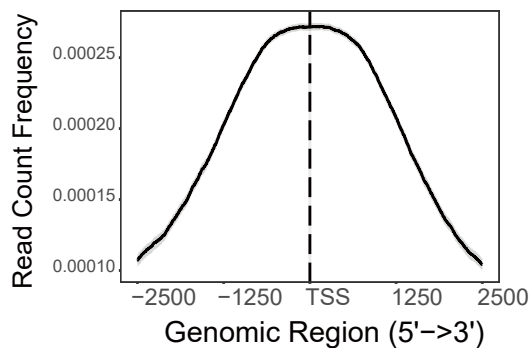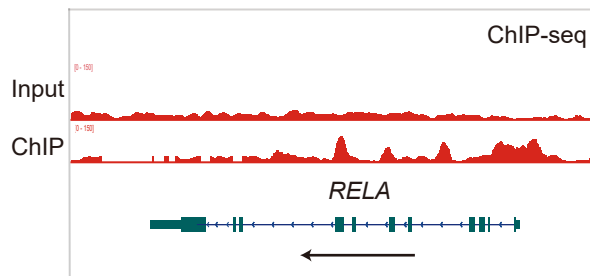

B

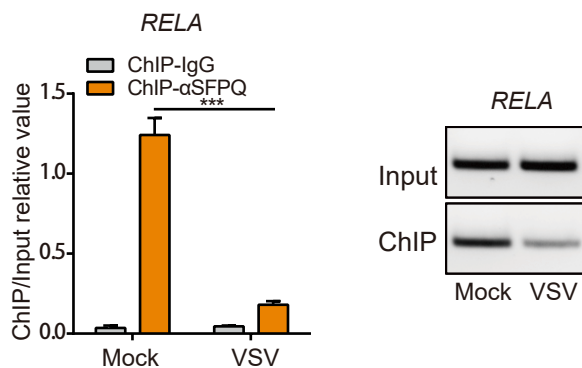

C

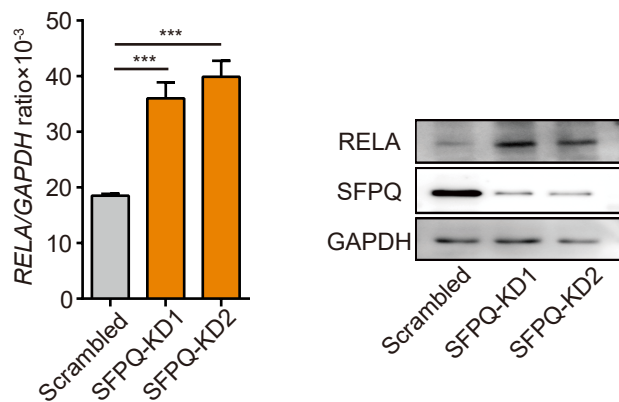

D

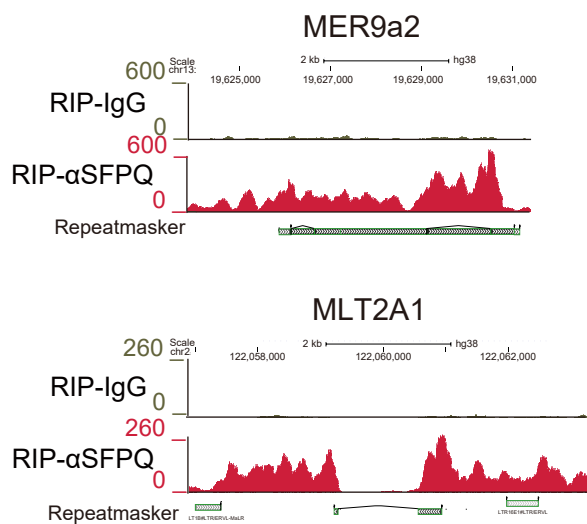

E

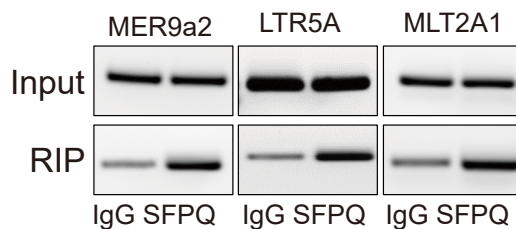

Supplement: FIG S5 [file mBio.00937-19-sf005.pdf]
