# Supplementary material for: Endogenous Retrovirus-Derived Long Noncoding RNA Enhances Innate Immune Responses via Derepressing RELA Expression
Source: mBio. 2019 Jul 30;10(4):e00937-19. doi: 10.1128/mBio.00937-19 (PMC6667616; doi:10.1128/mBio.00937-19)
Supplement: TABLE S1 [file mBio.00937-19-st001.docx]

**TABLE S1. Mass spectrometry analysis of SFPQ**

| **Sequence** | **Area** | **IonScore** | **Exp Value** | **MH+ [Da]** | **ΔM [ppm]** |
| --- | --- | --- | --- | --- | --- |
| ALAEIAKAELDDTPMR | 3367916 | 94.81 | 7.7E-09 | 1743.905 | 6.036834 |
| cSEGVFLLTTTPR | 1641072 | 77.27 | 4.54E-07 | 1480.753 | 4.618053 |
| FATHAAALSVR | 16160600 | 74.74 | 9.6E-07 | 1143.632 | 4.615511 |
| FGQGGAGPVGGQGPR | 62644684 | 72.82 | 1.05E-06 | 1341.673 | 4.93537 |
| LFVGNLPADITEDEFKR | 10906061 | 67.15 | 5.16E-06 | 1964.023 | 5.414469 |
| TEEKISDSEGFK | 6770373 | 66.58 | 2.96E-06 | 1369.656 | 5.53279 |
| AVVIVDDR | 36775053 | 61.34 | 2.43E-05 | 886.5036 | 4.892674 |
| SPPPGMGLNQNR | 11868396 | 60.7 | 1.89E-05 | 1267.628 | 5.017069 |
| SLDEMEKQQR | 4588321 | 58.89 | 2.38E-05 | 1263.606 | 4.84278 |
| ALAEIAKAELDDTPmR | 2255758 | 56.5 | 5.88E-05 | 1759.9 | 5.819406 |
| SLDEmEKQQR | 1092921 | 56.29 | 3.74E-05 | 1279.601 | 4.783817 |
| ISDSEGFK | 10324794 | 53.43 | 5.95E-05 | 882.4245 | 4.720869 |
| AVVIVDDRGR | 29541050 | 52.92 | 0.000122 | 1099.627 | 4.748422 |
| GIVEFASKPAAR | 35823102 | 52.76 | 0.000115 | 1245.701 | 4.543541 |
| FAQHGTFEYEYSQR | 13675257 | 51.33 | 7.58E-05 | 1762.791 | 4.927251 |
| GIVEFASK | 5593446 | 45.01 | 0.000803 | 850.4715 | 5.340386 |
| AELDDTPMR | 4497491 | 44.45 | 0.000357 | 1047.483 | 4.764035 |
| LFVGNLPADITEDEFK | 3224518 | 43.92 | 0.000989 | 1807.923 | 6.252325 |
| EEYEGPNKKPR | 2565799 | 41.04 | 0.001968 | 1346.676 | 4.357061 |
| EMQLRQEEER | 1132705 | 37.32 | 0.003309 | 1347.639 | 5.101058 |
| SLDEMEK | 2768801 | 37 | 0.002474 | 851.3868 | 6.185301 |
| EEEMMIR | 1648118 | 36.77 | 0.00305 | 937.4174 | 5.946868 |
| ALAEIAKAELDDTPmRGR | 2221901 | 35.43 | 0.007404 | 1973.023 | 5.446483 |
| RQREESYSR | 1744778 | 35.29 | 0.007942 | 1210.599 | 5.511218 |
| WKSLDEMEK | 1052260 | 33.08 | 0.012547 | 1165.562 | 5.22147 |
| EEEmmIR | 1833412 | 23.83 | 0.019872 | 969.4067 | 5.223344 |

**Sequence coverage of lnc-EPAV candidate binding protein (SFPQ)**

MSRDRFRSRGGGGGGFHRRGGGGGRGGLHDFRSPPPGMGLNQNRGPMGPGPGGPKPPLPPPPPHQQQQQPPPQQPPPQQPPPHQQPPPHQPPHQQPPPPPQESKPVVPQGPGSAPGVSSAPPPAVSAPPANPPTTGAPPGPGPTPTPPPAVPSTAPGPPPPSTPSSGVSTTPPQTGGPPPPPAGGAGPGPKPGPGPGGPKGGKMPGGPKPGGGPGMGAPGGHPKPPHRGGGEPRGGRQHHAPYHQQHHQGPPPGGPGPRTEEKISDSEGFKANLSLLRRPGEKTYTQRCRLFVGNLPADITEDEFKRLFAKYGEPGEVFINKGKGFGFIKLESRALAEIAKAELDDTPMRGRQLRVRFATHAAALSVRNLSPYVSNELLEEAFSQFGPIERAVVIVDDRGRSTGKGIVEFASKPAARKAFERCSEGVFLLTTTPRPVIVEPLEQLDDEDGLPEKLAQKNPMYQKERETPPRFAQHGTFEYEYSQRWKSLDEMEKQQREQVEKNMKDAKDKLESEMEDAYHEHQANLLRQDLMRRQEELRRMEELHSQEMQKRKEMQLRQEEERRRREEEMMIRQREMEEQMRRQREESYSRMGYMDPRERDMRMGGGGTMNMGDPYGSGGQKFPPLGGGGGIGYEANPGVPPATMSGSMMGSDMRTERFGQGGAGPVGGQGPRGMGPGTPAGYGRGREEYEGPNKKPRFStop

**Peptides identified in mass spectrometry analysis (highlighted in red).**
