# Supplementary material for: Endogenous Retrovirus-Derived Long Noncoding RNA Enhances Innate Immune Responses via Derepressing RELA Expression
Source: mBio. 2019 Jul 30;10(4):e00937-19. doi: 10.1128/mBio.00937-19 (PMC6667616; doi:10.1128/mBio.00937-19)
Supplement: TABLE S4 [file mBio.00937-19-st004.docx]

**TABLE S4.** **Primers information**

**1. Primers for** **qPCR.**

| Gene name | Strand | Primer sequence (5´to 3´) |
| --- | --- | --- |
| *Actin* | Forward | CATTGCTGACAGGATGCAGAAGG |
|  | Reverse | TGCTGGAAGGTGGACAGTGAGG |
| *Ifnb1* | Forward | GCCTTTGCCATCCAAGAGATGC |
|  | Reverse | ACACTGTCTGCTGGTGGAGTTC |
| *Il6* | Forward | TACCACTTCACAAGTCGGAGGC |
|  | Reverse | CTGCAAGTGCATCATCGTTGTTC |
| *Tnf* | Forward | GGTGCCTATGTCTCAGCCTCTT |
|  | Reverse | GCCATAGAACTGATGAGAGGGAG |
| *Rela* | Forward | TCCTGTTCGAGTCTCCATGCAG |
|  | Reverse | GGTCTCATAGGTCCTTTTGCGC |
| *Malat1* | Forward | TCGGTGCAAGGCTTAGGAAT |
|  | Reverse | TCTGCAGTATTGCATGTTAGGGA |
| *ACTIN* | Forward | CATTGCTGACAGGATGCAGAAGG |
|  | Reverse | TGCTGGAAGGTGGACAGTGAGG |
| *RELA* | Forward | TCCTGTTCGAGTCTCCATGCAG |
|  | Reverse | GGTCTCATAGGTCCTTTTGCGC |
| lnc-EPAV | Forward | AGGTCCTAGACCCACTTAGAGG |
|  | Reverse | TGAGCGTCCGCAAAACTGAAA |

**2. Primers for *in vitro* transcription and RNA pulldown.**

| Gene name | Strand | Primer sequence (5´to 3´) |
| --- | --- | --- |
| lnc-EPAV | Forward | GATCACTAATACGACTCACTATAGGGTTTCATTTGGTGCGTTGGCCG |
|  | Reverse | TGAAAGACCCTCGAGGGAAGACCCT |
| lnc-EPAV(E2) | Forward | GATCACTAATACGACTCACTATAGGGCGAGTCCCACCTCGCGTCTGGTCA |
|  | Reverse | GTTACCTTGGTCAGATTGTTGGGAC |

**3. Primers for RNA FISH.**

| Gene name | Strand | Primer sequence (5´to 3´) |
| --- | --- | --- |
| lnc-EPAV (sense) | Forward | AAAGGGGGGTGAAGAGAGAAGC |
|  | Reverse | GATCACTAATACGACTCACTATAGGGGATATTAAAGGAGGATTTATTAGATT |
| lnc-EPAV (antisense) | Forward | GATCACTAATACGACTCACTATAGGGCTATGCTGACCACTTCCTTTCAGATCCT |
|  | Reverse | CAGCTCTCACAGCTGGCTGCAAC |

**4. Primers for** **ChIP-qPCR and ChIP-PCR analysis.**

| Gene name | Strand | Primer sequence (5´to 3´) |
| --- | --- | --- |
| lnc-EPAV  promoter | Forward | TTCCTTGATAGGACATGACTCCTT |
|  | Reverse | GCCAGAATTTTTCACAGGCTTATATAG |
| *Rela*  promoter | Forward | CCCACACTCAATCTGCACACC |
|  | Reverse | ACCAGCTAGGGTCACACCGTG |
| *RELA*  promoter | Forward | GGTTAGGAAACCCGCGCGGA |
|  | Reverse | GTGGGTCCGCCGATTACTCACTT |

**5. shRNA target sequences**

| Gene name | Type | Primer sequence (5´to 3´) |
| --- | --- | --- |
| lnc-EPAV-sh1 | shRNA | GTTCTGTCAGAGGAATCTACG |
| lnc-EPAV-sh2 | shRNA | ATAGATCATTATTATAAACAC |
| *Sfpq*-sh | shRNA | ATGCGCCTTAATTCTTCCTGG |
| *SFPQ*-sh1 | shRNA | ATACATTGGATTCTTCTGGGC |
| *SFPQ*-sh2 | shRNA | TAGATTCCCAACAAACAACCG |
| Scrambled | shRNA | CAACAAGATGAAGAGCACCAA |

**6. sgRNA target sequences for CRISPR genomic engineering**

| Gene name | Type | Primer sequence (5´to 3´) |
| --- | --- | --- |
| lnc-EPAV-sg1 | sgRNA | GGCAGAGCAGAGTTCTTCGT |
| lnc-EPAV-sg2 | sgRNA | GCTCAGGCGTTGCTCCTCCG |

**7. Primers for the genotype of lnc-EPAV knockout mice**

| Gene name | Strand | Primer sequence (5´to 3´) |
| --- | --- | --- |
| lnc-EPAV-Mut-350bp | Forward | GCCCAAGATTACCCAGCCAGAATGT |
|  | Reverse | GGTTTCCAGAGCTTCCTGGCTTCTT |
| lnc-EPAV-WT-500bp | Forward | GCCCAAGATTACCCAGCCAGAATGT |
|  | Reverse | AAGGGTTGCATGCAGCATTTGGAAG |
