# Supplementary material for: Endogenous Retrovirus-Derived Long Noncoding RNA Enhances Innate Immune Responses via Derepressing RELA Expression
Source: mBio. 2019 Jul 30;10(4):e00937-19. doi: 10.1128/mBio.00937-19 (PMC6667616; doi:10.1128/mBio.00937-19)
Supplement: TABLE S5 [file mBio.00937-19-st005.docx]

**TABLE S5.** **Analysis of sgRNAs putative off-target sequences**

| No | sgRNA1 off-target Sequence | Chr | Position | Strand | Mismatches |
| --- | --- | --- | --- | --- | --- |
| 1 | GGCAGAGCAcAGTTCTgCGgTGG | 3 | 124394135 | - | 3 |
| 2 | GGCAGAGCAGAGTTCaTtGcTGG | 5 | 148867752 | - | 3 |
| 3 | GGCAtAGCtGtGTTCTTCGTGGG | 1 | 51416583 | + | 3 |
| 4 | GGCAGAGgAGAGTTaTTtGTAGG | 1 | 131092450 | - | 3 |
| 5 | aGCAGAGCAGAGacCTTCGTTGG | 13 | 75930101 | + | 3 |
| 6 | GGtAGAGCAGAGTgCTTCcTGGG | 9 | 110690240 | - | 3 |
| 7 | GGCAGAaCAcAGTTCTTtGTCGG | 11 | 52468960 | - | 3 |
|  |  |  |  |  |  |
| No | **sgRNA2 off-target Sequence** | **Chr** | **Position** | **Direction** | **Mismatches** |
| 1 | GCTCAGGCtTTGCTgCTCCaGGG | 13 | 50187201 | + | 3 |
| 2 | GCTCAGGCtTTGCTgCTCCaGGG | 13 | 50623944 | - | 3 |
| 3 | tCTgAGcCGTTGCTCCTCCGGGG | 6 | 34160355 | - | 3 |
| 4 | tCTCAGGCGTTtCTtCTCCGTGG | 18 | 65868240 | - | 3 |

The Sanger Sequencing results were shown in below (The sgRNA putative off-target sequences were labeled in dark blue background):

UCSC(Mm10): TAGTTCCATTGGTTCATTTCCACCGCAGAACTGTGCTCTGCCCAGGCCTGTCTTCAATGCT
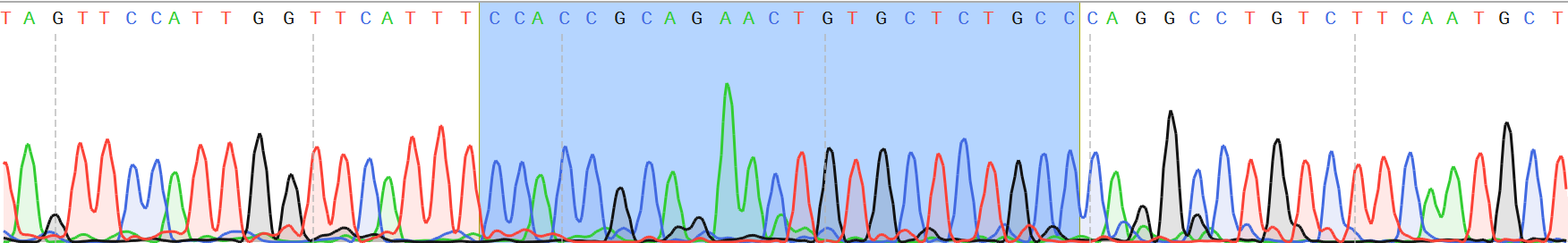


sgRNA1-1

UCSC(Mm10): AAGTTGTGGCTTGGACCGGGCAGAGCAGAGTTCATTGCTGGGCATCCCCCCACTCCCGA
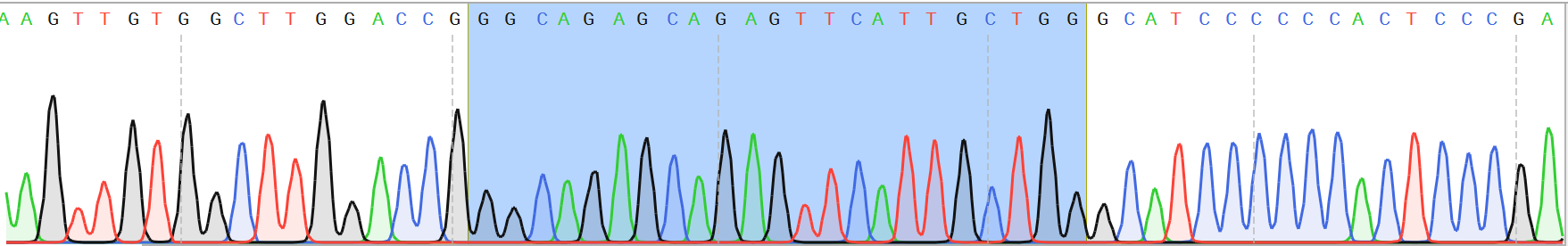


sgRNA1-2

UCSC(Mm10): ACTGGTCTAATGCATGTGGGCATAGCTGTGTTCTTCGTGGGAGAGACCTAGTCCATATGA
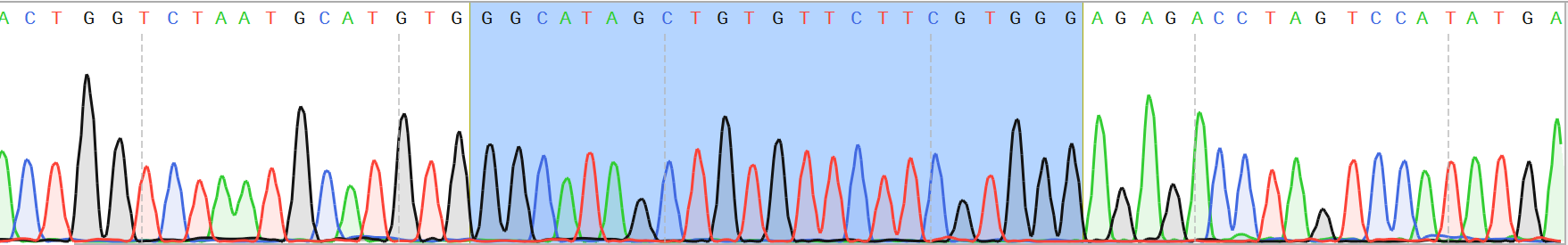


sgRNA1-3

UCSC(Mm10): TCAAGAGCTGTTGCAGTCCCTACAAATAACTCTCCTCTGCCCACTCACCTCCATCCAAGG
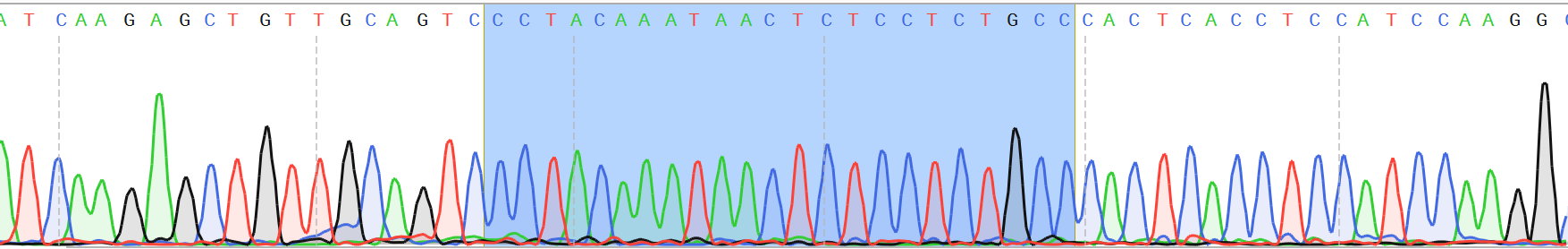


sgRNA1-4

UCSC(Mm10): CAGCCTCGGTATGGATGCCAGCAGAGCAGAGACCTTCGTTGGGGGCTGCACTAGGATCCTT
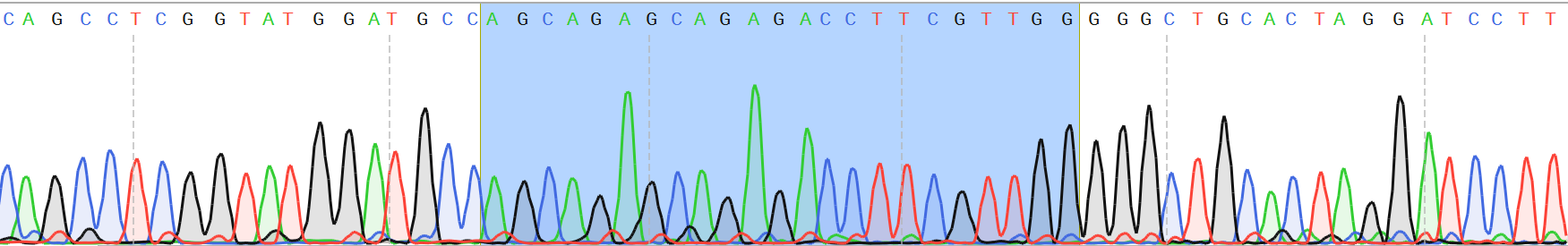


sgRNA1-5

UCSC(Mm10):CCTGCTCTACCACCTCTAACCCAGGAAGCACTCTGCTCTACCACGTCTAGCTCAGGAGCA
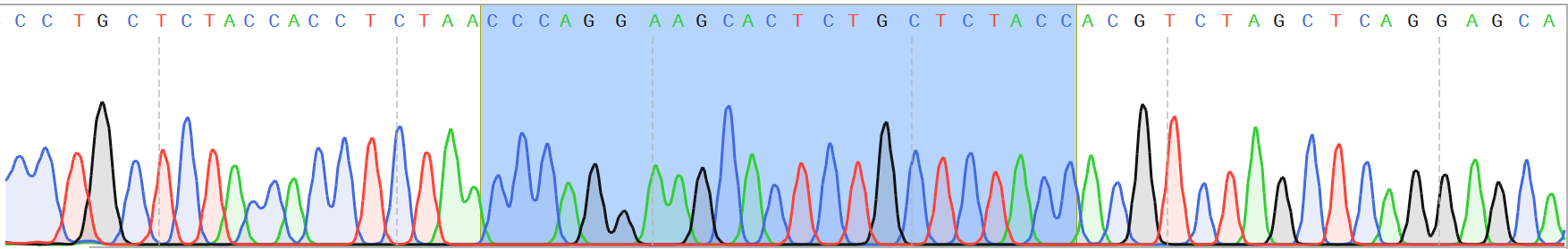


sgRNA1-6

UCSC(Mm10):TAGTTGGGAGGAGAAAGACCGACAAAGAACTGTGTTCTGCCAAGCAGTGCAGGCACAGA
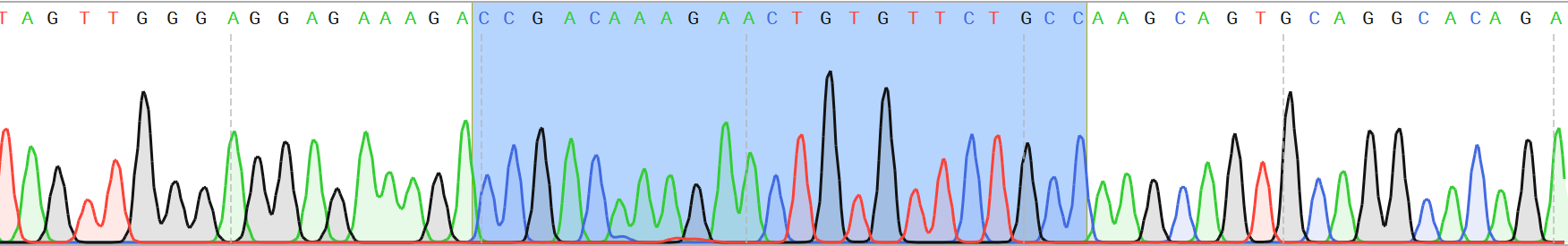


sgRNA1-7

UCSC(Mm10):GGGCGGCCTGGACAATGGCCCTGGAGCAGCAAAGCCTGAGCTTTGCTAAGCTGCTCTGT
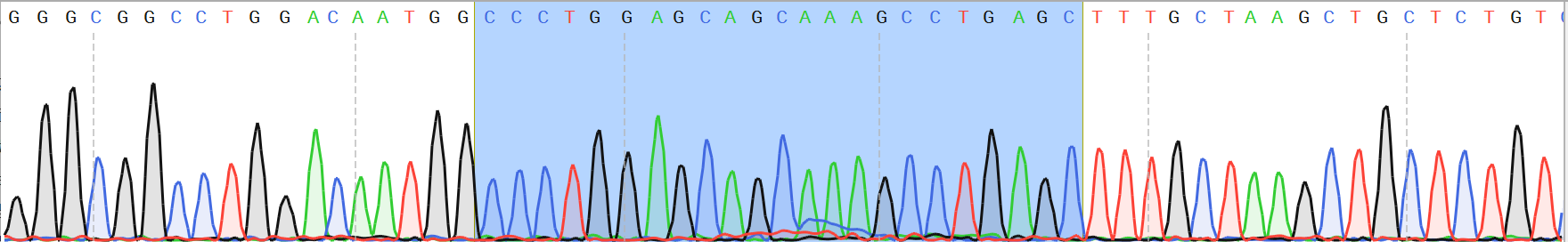


sgRNA2-1

UCSC(Mm10):GACAGAGCAGCTTAGCAAAGCTCAGGCTTTGCTGCTCCAGGGCCATTGTCCAGGCCGCCCG
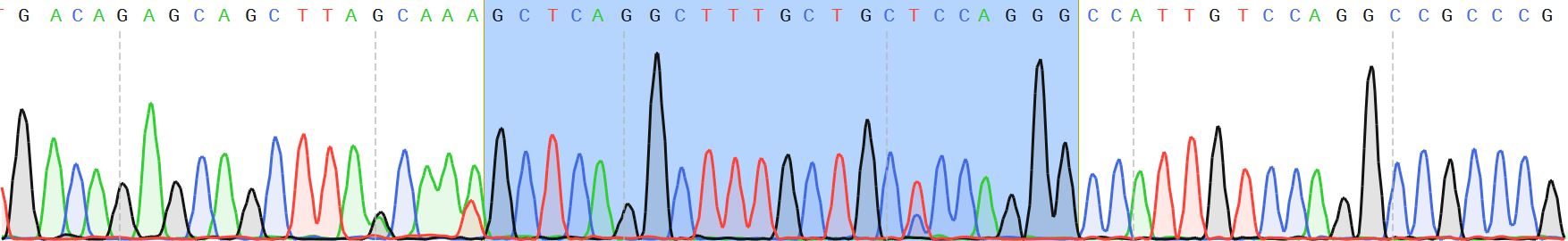


sgRNA2-2

UCSC(Mm10):TAACAGCCCTGTTTGGACGCCCCGGAGGAGCAACGGCTCAGACAAACATGAGATAAGCCGT
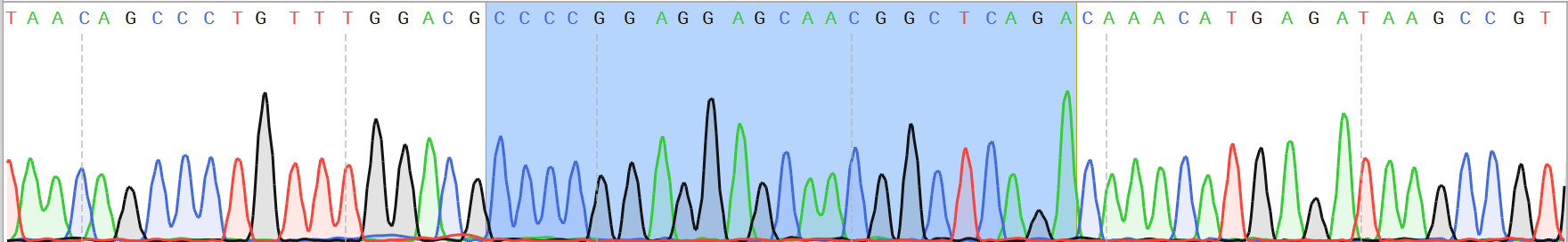


sgRNA2-3

UCSC(Mm10):ATTGCATAAGAGAAAATAATCCACGGAGAAGAAACGCCTGAGACTTACCACACCAAACGTCAT
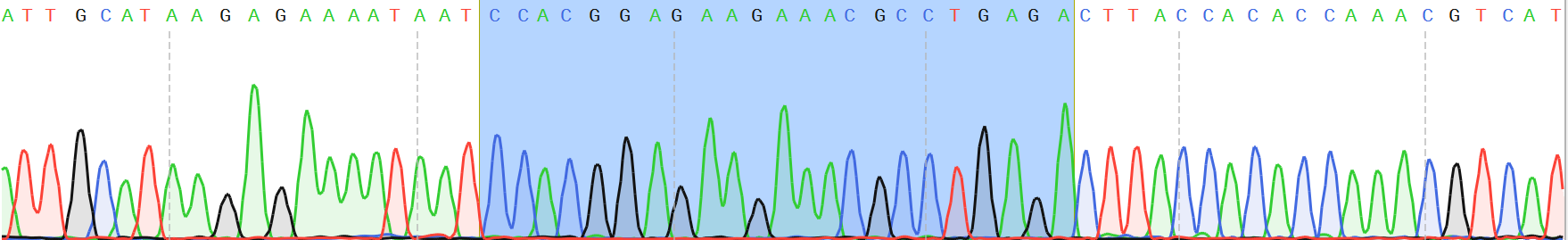


sgRNA2-4
